# Stability and genetic parameters for cassava yield attributes in the tropical humid region of Brazil

**DOI:** 10.1007/s10681-024-03384-5

**Published:** 2024-07-19

**Authors:** Juraci Souza Sampaio Filho, Marcos de Souza Campos, Eder Jorge de Oliveira

**Affiliations:** 1 Centro de Ciências Agrárias, Ambientais e Biológicas, Universidade Federal do Recôncavo da Bahia, Cruz das Almas, BA 44380-000 Brazil; 2 Embrapa Mandioca e Fruticultura, Nugene, Cruz das Almas, BA 44380-000 Brazil

**Keywords:** Correlation, Multi-environments, Genotype vs environment interaction, Breeding, Cultivar

## Abstract

The performance differences in cassava genotypes arising from genotype vs. environment interactions (G × E) often lead to responses that are significantly lower than expected for selection. The objective of this study was to evaluate different stability methods, both parametric and non-parametric, such as additive main-effects and multiplicative interaction (AMMI), main effect of genotypes plus G × E (GGE), and weighted average of absolute scores (WAASB), in order to quantify the G × E in multi-environmental trials. A total of 12 genotypes were assessed across 12 environments using a completely randomized block design, with three replicates for traits such as fresh root yield (FRY) and dry matter content in the roots (DMC). The data were subjected to analysis of variance and the Scott Knott test (*p* < 0.05). The sum of squares (SQ) of genotypes, environment, and G × E effects were equally distributed for FRY, whereas for DMC, these effects accounted for 64.1%, 21.9%, and 13.8% of the SQ, respectively, indicating a lower environmental effect on this characteristic. Using the AMMI, GGE, and WAASB methods, genotypes with high agronomic performance and stability for FRY (BR11-34–41 and BR11-34–69) (> 32 t ha^−1^) and DMC (BRS Novo Horizonte, BR12-107–002, and BR11-24–156) (> 37%) were identified. The broad-sense heritability (${h}^{2}$) for FRY and DMC was estimated to be 0.45 and 0.75, respectively. Approximately 72% of the methods identified BRS Novo Horizonte as the genotype with the highest stability and performance for DMC, while 47% identified genotypes BR11-34–41 and BR11-34–69 for FRY and intermediate DMC. Genotype BR11-24–156 exhibited high static stability according to 50% of the methods. Significant correlations were observed between stability and agronomic performance across the different methods, enabling the formation of groups based on stability concepts. Additionally, it was found that two mega-environments existed for FRY, whereas DMC displayed a single mega-environment with similar patterns, indicating an absence of G × E. We identified superior genotypes that could be promoted to national performance trials to develop stable cultivars with better yield attributes in cassava.

**Supplementary Information:**

The online version contains supplementary material available at 10.1007/s10681-024-03384-5.

## Introduction

Cassava (*Manihot esculenta* Crantz) plays a crucial role as a carbohydrate source for millions of people in various countries, particularly those with a tropical climate. This versatile plant is commercially exploited for all its parts, with the tuberous roots being the main focus due to their starch content. In fact, cassava is the second most significant global source of starch (Stapleton 2012; Ceballos et al. 2020), finding applications in human food, drug production, biopolymers, fuel, and more.

Over the past decade, the worldwide production of cassava has witnessed a remarkable growth of over 27%. This increase can be attributed to several factors, including advancements in cultivation systems and the utilization of genetically improved varieties with enhanced root and starch productivity (Akinwale et al. 2010; Faostat 2020; Oliveira et al. 2020a). Currently, global cassava production stands at approximately 302.66 million tons, cultivated across 28.24 million hectares (Faostat 2020). Among the top four cassava producers worldwide, Nigeria, Thailand, Indonesia, and Brazil, the production of fresh roots reaches 60.00, 28.99, 18.30, and 18.20 million tons, respectively.

Cassava cultivation spans across multiple countries, primarily in regions located between latitudes of 30° N and 30° S. The cultivation of cassava faces diverse edaphoclimatic conditions, characterized by significant variations in factors such as rainfall, temperature, soil attributes, and sunlight availability (El-Sharkawy 2007). In this context, the varietal component assumes great importance, as the choice of variety is a critical agricultural input. Regardless of the farmer's technological level, the selected variety must effectively utilize these factors to express its genetic potential and ensure high yields.

The process of developing and recommending a new cassava variety for planting is time-consuming, typically taking between 8 to 10 years. Additionally, there are considerable costs associated with phenotyping in various environments during conventional breeding (Oliveira et al. 2014; Ceballos et al. 2015; Wolfe et al. 2017; Andrade et al. 2019). In the initial five years, a large number of genotypes are evaluated with few repetitions and in a limited number of environments. Subsequently, in the last three years, experiments known as value of cultivation and use (VCU) trials are conducted with a smaller set of genotypes. These trials typically involve three or four replications and are conducted across a wide range of environments. This approach allows for a detailed exploration of the genotypes × environments (G × E) interaction, enabling a comprehensive assessment of adaptability and stability parameters. These parameters play a crucial role in recommending the best-performing clones.

The G × E arises from the inconsistent phenotypic expression of traits when genotypes are exposed to different environmental conditions. This inconsistency leads to a reduction in the correlation between the phenotypic and genotypic values of evaluated genotypes, which is a crucial factor to consider when selecting and recommending cultivars with high genetic potential (Yan and Holland 2010). However, G × E can also be beneficially exploited to identify genotypes with greater stability and adaptability across specific environments of interest (Tumuhimbise et al. 2014). In general, the adaptability of a genotype refers to its ability to respond favorably to a particular environment, while stability relates to the predictability of the genotype's agronomic performance across a range of environments (Finlay and Wilkinson 1963; Eberhart and Russell 1966). According to these authors, the genotype's average performance compared to the overall average of all tests determines the favorable and unfavorable environments. This information is valuable for regionalized recommendations of new clones.

Several studies have demonstrated the presence of G × E in cassava (Maroya et al. 2012; Agyeman et al. 2015; Mtunguja et al. 2016; Adjebeng-Danquah et al. 2017; Chipeta et al. 2017; Nduwumuremyi et al. 2017; Fotso et al. 2018; Masinde et al. 2018; Jiwuba et al. 2020). Therefore, it is important for research studies to identify specific clones that perform well in each environment or exhibit general adaptability. To interpret and utilize G × E, various parametric and non-parametric statistical models have been developed. In general, the additive main effects and multiplicative interaction (AMMI) and genotype main effects plus genotype × environment interaction effects (GGE) methodologies have been widely employed. These methods capture the most significant portion of the sum of squares of G × E interaction for agronomic traits related to the performance of elite genotypes, disease resistance, and root quality in the final evaluation stages (Kvitschal et al. 2009; Tumuhimbise et al. 2014; Esuma et al. 2016; Agustina et al. 2020; Peprah et al. 2020).

The AMMI method combines analysis of variance (ANOVA) for the additive main effects of genotypes and environments with principal component analysis (PCA) to assess the multiplicative interaction effects of G × E (Gauch and Zobel 1996 This approach enhances the model's predictive capacity and is particularly useful for identifying specific adaptations between genotypes and environments. However, the AMMI method lacks discriminative capacity and the ability to represent biplot graphs due to the absence of mathematical properties of vector products (Gauch and Zobel 1996, Gauch Jr 2013). On the other hand, the GGE method does not separate the genotype effect from the G × E interaction. By considering the combined value of G + G × E, this method captures the total genetic variation rather than exclusively focusing on the G × E component. It also allows for the identification of the performance of the best genotypes, mega-environments, and heterotic groups (Yan 2016).

Various authors have utilized AMMI and/or GGE methods for analyzing G × E data in cassava, recognizing their unique characteristics and complementary nature (Alwala et al. 2010; Frutos et al. 2013; Noerwijati et al. 2014; Nduwumuremyi et al. 2017). However, some researchers suggest combining multivariate methods like AMMI and GGE with other complementary methodologies such as mixed models, uniparametric approaches, and nonparametric approaches to fully explore the sum of squares of the G × E interaction and enhance the reliability of results. This is particularly important due to the lack of agreement among different methods in identifying genotypes with high agronomic performance and stability or adaptability to specific environments.

One example of a complementary approach is the use of the harmonic mean of predicted genotypic values (HMGV) and the weighted mean of absolute scores (WAASB), which simultaneously select genotypes based on stability and yield parameters (Olivoto et al. 2019). Despite its crucial importance in selecting and recommending new genotypes for cultivation in specific target regions, research on the complementarity of methods for evaluating the G × E interaction in cassava has been limited. The objectives of this study were: i) to estimate the adaptability and stability of cassava genotypes evaluated in multi-environment trials (MET) for subsequent planting recommendations; ii) to explore the correlation between parametric methods (univariate and multivariate), mixed models, and nonparametric models in estimating the G × E interaction; and iii) to identify the methods that provide the best estimation of stability and adaptability in cassava, considering dynamic and static stability as well as the magnitude and significance of G × E effects.

## Material and methods

### Variety value for cultivation and use tests (VCU)

**Fig. 1:**
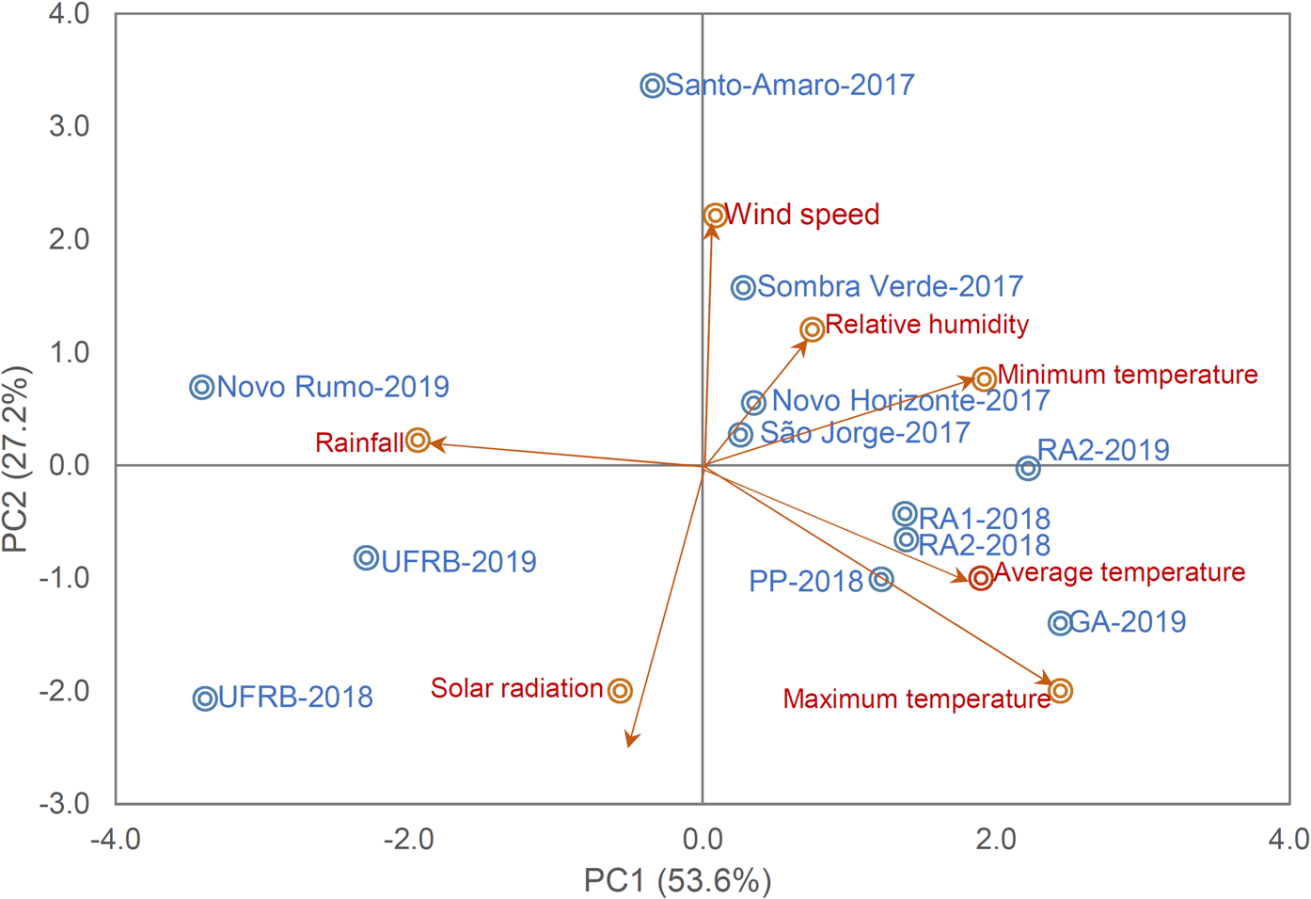
Scatterplot of the first two principal components of seven environmental (climatic) variables and 12 environments used in the variety value for cultivation and use (VCU) trials. Maximum, minimum and average temperature (Tmax, °C, Tmin, °C and Tav, °C, respectively), rainfall (Rain, mm day^−1^), relative humidity (Rh, %), wind speed (S/wind, ms^−1^) and solar radiation (Sol/rad, MJ/m^−2^ day^−1^)

The VCU trials in multi-environment trials (METs) were conducted by Embrapa Mandioca e Fruticultura over three harvests in the crop’s seasons of 2017, 2018, and 2019. Each year, the experiments were set up in four different locations, resulting in a total of 12 environments in the state of Bahia, which is characterized as a tropical hot and humid region. According to the Köppen classification, the predominant climate in the region is Aw and Am. Rainfall is concentrated between the months of April and July, with an average of 1100 mm per year, irregularly distributed, and a prolonged dry period lasting 5–7 months. During the period from planting to harvesting, meteorological data including temperature, precipitation, and humidity were collected by interpolating data from the three closest automatic meteorological stations: Amargosa, Cruz das Almas, and Valença-Bahia (INMET 2020) (Fig. 1). Descriptions of the test sites are provided in Table 1.

**Table 1:** List of the 12-variety value for cultivation and use (VCU) trials carried in the tropical humid region of Brazil

City	Location	Year	Coordinates	Ground	Code
Laje (BA)	Novo Horizonte	2017	13°09′52″S, 39°25′59″W	Red-Yellow Argissolo	NH-2017
Santo Amaro (BA)	Santo Amaro	2017	12°34′23’’S, 38°42′53’’W	Chrome Vertissolo	SA-2017
Laje (BA)	Sombra Verde	2017	13°08′36″S, 39°25′46″W	Yellow Latosol	SV-2017
Laje (BA)	São Jorge	2017	13°09′52″S, 39°25′59″W	Red-Yellow Latosol	SJ-2017
Cruz das Almas (BA)	UFRB	2018	12°39′11″S, 39°07′19″W	Yellow Dystrophic Latosol	UFRB-2018
Laje (BA)	PP	2018	13°09′52″S, 39°25′59″W	Yellow Latosol	PP-2018
Laje (BA)	Rio de Areia 1	2018	13°08′47″S, 39°17′58″W	Yellow Latosol with sandy texture	RA1-2018
Laje (BA)	Rio de Areia 2	2018	13°09′52″S, 39°25′59″W	Medium-textured yellow Latosol	RA2-2018
Cruz das Almas (BA)	UFRB	2019	12°39′11″S, 39°07′19″W	Yellow Dystrophic Latosol	UFRB-2019
Valença (BA)	Novo Rumo	2019	13°22′13″S, 39°04′23″W	Hydromorphic Spodosol	NR-2019
Laje (BA)	Gavião	2019	13°09′52″S, 39°25′59″W	Red-Yellow Latosol	GA-2019
Laje (BA)	Rio de Areia 2	2019	13°09′52″S, 39°25′59″W	Medium-textured yellow Latosol	RA2-2019

The VCU trials involved 12 genotypes selected from the cassava breeding program, including six clones in the final validation phase and six control varieties (Table 2). Soil preparation for cultivation followed conventional practices, including weed desiccation, plowing, and harrowing to incorporate crop residues. A cassava planter was used to open planting furrows and apply fertilizers based on soil analysis. Planting was done manually using standard cuttings measuring 18–20 cm obtained from 12-month-old stems that were free of pests and diseases. The cuttings were placed horizontally along the planting line.

**Table 2:** Description of the genotypes evaluated in 12-variety value for cultivation and use (VCU) trials evaluated in the crop value and use trials

Genotypes	Year	Maturity-cycles	Origin
New clones			
BR11-24–156	2020	12 months	CNPMF-BRA
BR11-34–41	2020	12 months	CNPMF-BRA
BR12-107–002	2020	12 months	CNPMF-BRA
BR11-34–64	2020	12 months	CNPMF-BRA
BR11-34–45	2020	12 months	CNPMF-BRA
BR11-34–69	2020	12 months	CNPMF-BRA
Checks			
BRS Novo Horizonte	2018	12 months	CNPMF-BRA
BRS Formosa	2002	12 months	CNPMF-BRA
Corrente	–	12–18 months	DP-BRA
IAC-90	–	12–18 months	IAC-BRA
Cigana Preta	2002	12–18 months	CNPMF-BRA
BRS Mulatinha	2005	12 months	CNPMF-BRA

**CNPMF Embrapa Mandioca e Fruticultura, *IAC* Insituto Agronomico de Campinas, *DP* Public Domain, *BRA* Brazil

The VCU trials were arranged in a completely randomized block design with three replications. Each plot consisted of four rows with 25 plants each, spaced at 0.90 m between rows and 0.80 m between plants. Cultural treatments after planting followed the recommended practices for cassava cultivation (Souza et al. 2006).

## Traits evaluated

At 12 months after planting, the following traits were evaluated: 1) root yield (FRY, in t ha^−1^) considering the weight of all roots in the plot, and then adjusted to tons per hectare; and 2) root dry matter content (DMC, in %) obtained from the gravimetric methodology (Kawano et al. 1987).

## Individual and joint analysis

The data obtained from the VCU trials were subjected to individual analysis of variance (ANOVA) to assess the significance of different factors. The homogeneity of residual variances was tested using Bartlett's test. The joint analysis of variance was then performed without restrictions, ensuring that the ratio between the highest and lowest mean square value of the residue was less than 7, as suggested by Pimentel-Gomes (2009).

For the individual ANOVA, the ea1 and ea2 functions of the easyanova package (Arnhold et al. 2013) in the R software version 4.2.0 (R Core Team 2022) were utilized. The statistical model employed for individual analysis is represented by the equation: ${\text{y}}_{\text{ij}} =\upmu + {\text{g}}_{\text{i}} +{\text{b}}_{\text{j}}+ {\text{e}}_{\text{ij}}$. Here, ${y}_{ij}$ denotes the vector of phenotypic values associated with genotype *i* in block *j*; $\upmu$ is the overall mean; ${\text{g}}_{\text{i}}$ is the effect of genotype *i*; ${\text{b}}_{\text{j}}$ is the effect of block *j*; and ${\text{e}}_{\text{ij}}$ is the experimental error associated with the plot receiving genotype *i* in block *j*.

The joint analysis of variance was performed using the AMM" function of the agricolae package (Mendiburu 2021). The statistical model for the joint analysis is given by the equation: ${y}_{ijk}= \mu + {\alpha }_{i}+{\tau }_{j}+ \sum_{k=1}^{k}{\lambda }_{k}{{\alpha }_{ik}{t}_{jk}}+{\rho }_{ij} + {\epsilon }_{ij}$. Here, ${y}_{ijk}$ represents the vector of phenotypic values of genotype i associated with block *j* within environment *k*; $\upmu$ s the overall mean; ${\alpha }_{i}$ is the main effect of genotype; ${\tau }_{j}$ is the main effect of the environment; ${\lambda }_{k}$ is the singular value for the axis of the principal component of the G × E interaction (IPCA), explaining the interaction of the data; ${\alpha }_{ik}$ is the i-th element of the k-th eigenvector; ${\tau }_{jk}$ is the j-th element of the k-th eigenvector; ${\rho }_{ij}$ represents the residue if not all IPCAs are used; and ${\varepsilon }_{ij}$ is the residual for genotype *i* in environment *j*, which is not explained by the model. The phenotypic means for each genotype and environment were then grouped using the Scott-Knott test implemented in R software version 4.2.0 (R Core Team 2022).

The experimental quality of the VCU tests was evaluated using the test accuracy values (${\widehat{r}}_{\widehat{gg}}$), which were calculated using the formula: ${\widehat{r}}_{\widehat{gg}}=\sqrt{1-\frac{1}{F}}$, where F is the ratio of the mean square of the genotype effects to the mean square of the G × E interaction effects. The test accuracy provides an indication of the reliability and precision of the experimental results.

## Variance components and genetic parameters

The variance components, including genotypic, phenotypic, environmental, and G × E interaction, were estimated using the mean squares obtained from the analysis of variance. The heritability for each trait was also estimated. The equations used for these estimations are as follows: ${h}^{2}=\frac{{\sigma }_{g}^{2}}{{\sigma }_{p}^{2}}$ and ${\sigma }_{p}^{2}=\left({\sigma }_{g}^{2}\right)+\left(\frac{{\sigma }_{gxe}^{2}}{E}\right)+\left(\frac{{\sigma }_{e}^{2}}{ER}\right)$: where ${\sigma }_{g}^{2}$ is the genetic variance; ${\sigma }_{p}^{2}$ is the phenotypic variance; ${\sigma }_{gxe}^{2}$ is the variance of the G × E interaction; ${\sigma }_{e}^{2}$ is the variance of the pooled error; $E$ the number of environments; and $R$ the repetition number.

The genotypic coefficients of variation ($CVG$), phenotypic ($CVP$) and residual $(CVr$) coefficients of variation were estimated according to Singh and Chaudhary (1979), where: $CVG \%=\sqrt{{\sigma }_{g}^{2}}/\mu \times 100$, $CVP \%=\sqrt{{\sigma }_{p}^{2}}/\mu \times 100$ and $CVr \%=\sqrt{{\sigma }_{e}^{2}}/\mu \times 100$, where $\mu$ is the overall mean. The expected genetic gain with selection (GA) was estimated using the formula: $GA=\left(K\right){\sigma }_{p} {h}^{2}$, where $GA$ is the expected genetic gain, *K* represents the selection differential (15% selection intensity), ${\sigma }_{p}$ is the phenotypic standard deviation.

The genetic advancement as a percentage of the mean (GAM) was estimated by the formula: $GAM=GA/\mu \times 100$, where $GAM$ is the genetic advancement as a percentage of the mean, $GA$ is the expected genetic gain, $\mu$ represents the overall mean. The $CVratio$ was obtained by calculating the ratio between $\frac{CVG}{CVr}$, and the $P-G$ was obtained by calculating the difference between $CVP$ and $CVG$. These calculations and estimations were performed using the "metan" package (Olivoto and Lúcio 2020) in the R software version 4.2.0 (R Core Team 2022).

## Adaptability and stability analysis

The adaptability and stability parameters were estimated using various statistical models, including univariate and multivariate parametric models, as well as mixed and non-parametric models (Table 3). The agricolae package (Mendiburu 2021) was used for the (AMMI) and (ASV) analyses, while the GGEBiplotGUI package (Frutos et al. 2013) was used for the GGE analysis. The metan package (Olivoto and Lúcio 2020) was employed for other methods.

**Table 3:** Methods employed to quantify the genotype x environment interaction and determine adaptability and stability parameters based on parametric, nonparametric, multivariate models, as well as the linear mixed models’ approach

	Methods	^#^Abr	*Parameters	Reference	Statistical Models
Univariate Parametric	Standard Deviation	$DP$	E	Karl Pearson (1894)	$DP=\sqrt{{\sum }_{i=1}^{n}{{(X}_{i}-{M}_{A})}^{2}/n}$
	Confidence index—general environment	$li\left(g\right)$	A / E	Annicchiarico (1992)**	$Iig= \overline{\text{Y} }ig-{Z}_{(l-\alpha )} \left(\sigma ig\right)$
	Confidence index—favorable environment	$li\left(f\right)$	A / E	Annicchiarico (1992)**	$Iif= \overline{\text{Y} }if-{Z}_{(l-\alpha )} \left(\sigma if\right)$
	Confidence index—unfavorable environment	$li\left(d\right)$	A / E	Annicchiarico (1992)**	$Iid= \overline{\text{Y} }id-{Z}_{(l-\alpha )} \left(\sigma id\right)$
	Superiority index	$Pi$	A	Lin and Binns (1988)	$Pi={\sum }_{\text{j}=1}^{\text{n}}{\left(\text{Xij}-\text{Mj}\right)}^{2}/2n$
	Regression coefficient	${R}^{2}$	A	Eberhart; Russell (1966)	${R}^{2}=\sum j \left({{Y}_{ij}-\overline{Y} }_{i.}\right)\left({\overline{Y} }_{.j}- \overline{Y }..\right)/\sum j{\left({\overline{Y} }_{.j}- \overline{Y }..\right)}^{2}$
	Regression variance	$${S}_{di}^{2}$$	E	Eberhart; Russell (1966)	$$Dji = \frac{{\sum i .j Y_{ij} - sy_{i}^{2} + bi \sum j \underset{\raise0.3em\hbox{$\smash{\scriptscriptstyle\cdot}$}}{{\hat{\text{e}} }}j)}}{s - 2} - \frac{CME}{r}$$
	Square root of the mean square error	RMSE	E	Eberhart; Russell (1966)	$\text{RMSE}=\sqrt{{\sum }_{i=1}^{n}{\left(0-P\right)}^{2}/n}$
	Weighted mean of absolute scores	WAASB	A / E	Olivoto et al. 2019	$WAASB={\sum }_{k=1}^{p}{IPCA}_{ik} x {EP}_{k}/{\sum }_{k=1}^{p}{EP}_{k}$
	Regression coefficient	$Bi$	A	Perkins; Jinks (1968)	$Bi=\sum j \left({{Y}_{ij}-\overline{Y} }_{i.}-{\overline{Y} }_{.j}+ \overline{Y }..\right)\left({\overline{Y} }_{.j}- \overline{Y }..\right)/\sum j{\left({\overline{Y} }_{.j}- \overline{Y }..\right)}^{2}$
	Variance of regression variance	$$Di$$	E	Perkins; Jinks (1968)	$$Dji = \sum i .j Y_{ij} - sy_{i.}^{2} + bi \sum j \underset{\raise0.3em\hbox{$\smash{\scriptscriptstyle\cdot}$}}{\hat{e}} j/s - 2$$
	Ecovalence	$Wi$	A / E	Wricke (1962)	$Wi={\left({{Y}_{ij}-\overline{Y} }_{i.}-{\overline{Y} }_{j.}+ \overline{Y }..\right)}^{2}$
	Variance of environment	${S}^{2}x$	A / E	***Roemer (1917)	${S}^{2}x=\sum j{\left(rij-\overline{ri }.\right)}^{2}/s-1$
Non-parametric	Average absolute rank difference of genotype on the environment	${Si}^{1}$_,_ ${Si}^{6}$	A / E	Nassar; Huehn (1987)	$Si^{1} = 2\mathop \sum \limits_{j}^{n - 1} \frac{{\mathop \sum \nolimits_{j = j + 1}^{n} rij - rij^{\prime } }}{{\left[ {n\left( {n - 1} \right)} \right]}}$ e ${Si}^{6}={\sum }_{j=1}^{n}\lceil\frac{rij-\stackrel{-}{ri.}}{\stackrel{-}{ri.}}\rceil$
Parametric Multivariate	ASV stability value of AMMI	$ASV$	A / E	Purchase et al. (2000)	$ASV={\sqrt{\left[\frac{IPCA1 }{IPCA2 }IPCA1\right]}}^{2}+{IPCA1}^{2}$
	Genotype main effect plus genotype by environment interaction	$GGE biplot$	A / E	Yan (2002)	$Yij=\mu +\alpha i+\beta j+\phi ij+\varepsilon ij$
Mixed model	Harmonic mean of genetic values	$HMGV$	E	Resende (2007a)	$MHVG= \text{E}/\sum_{J=1}^{E}\frac{1}{Gvij}$

*A adaptability, *E* stability

**Modified by Schmildt and Cruz (2005)

***cited by Becker; Léon (1988)

The multivariate parametric analyses GGE and AMMI (ASV) were represented using two-dimensional graphs based on genotypes and environmental scores, represented by PC1 (phenotype of the trait under analysis) and PC2 (stability parameter). The GGE biplots were constructed using the method (centralization = 2) (scale = 0) and (PVS = 2), which represent performance, stability, and adaptability. The AMMI value (ASV) was used to quantify and classify genotypes based on performance stability. The ASV is determined by the distance from the coordinate point to the origin of the biplot, i.e., the ratio between the sum of squares on the PC1 axis and the sum of squares on the PC2 axis. A smaller ASV value indicates a more stable genotype (Purchase et al. 2000).

Simultaneous selection between performance and stability, based on the mixed linear model (LMM), employed two criteria: the harmonic mean of predicted genotypic values ($HMGV$) and the weighted mean of absolute $WAASB$ scores. A higher $HMGV$ indicates a lower standard deviation of genotype behavior in different environments. Thus, the genotype with the highest $HMGV$ in the evaluation environments received rank 1, up to the gth genotype, and was considered the most stable. In the case of $WAASB$, the genotype with the lowest mean received rank 1, up to the gth genotype.

Subsequently, the genotypes were classified based on different adaptability and stability parameters using the phenotypic values of each variable under study. For methods based on variance estimates ($DP$, ${S}^{2}d$, $Di$, $Wi$, and ${S}^{2}x$), lower values indicate more stable genotypes. Conversely, for the ${R}^{2}$ parameter of Eberhart and Russel and $Bi$ of Perkins and Jinks, values close to 1 indicate more adapted genotypes. Therefore, linear regression coefficients ($bi$ and $Bi$) equal to 1 and non-linear regression deviation (${S}^{2}d$ and $Di$) equal to zero received rank 1 as the best genotype, up to the gth genotype.

The Annicchiarico method involves the calculation of the confidence index for the general environment $li\left(g\right)$, with decomposition for favorable environment $li\left(f\right)$ and unfavorable environment $li\left(d\right)$. Positive values of the index indicate favorable environments, while negative values indicate unfavorable environments for cultivation. The average performance of the genotype compared to the overall average of all tests determines whether an environment is favorable or unfavorable. Genotypes with higher parameter values are ranked higher, with rank 1 assigned to the genotype with the highest parameter value.

The nonparametric method of Nassar and Huehn (1987) considers the classification of genotypes in each evaluation environment. A genotype is considered stable if its classification remains relatively constant across multiple evaluation environments, indicating low environmental variation. The stability parameters include ${Si}^{1}$, which is the average absolute differences in genotype classification across environments, and ${Si}^{6}$, which is the sum of squares of genotype classifications relative to the mean classification across environments. Genotypes with lower values of ${Si}^{1}$ and ${Si}^{6}$ are considered stable. Genotypes are ranked based on these stability indices. Rankings of the traits were also calculated using the Scott-Knott test, where the genotype with the highest value receives rank 1, and so on until the gth genotype.

To assess the correlations between stability and adaptability methods, Spearman's correlation coefficients (rs) were estimated using a matrix of ranks obtained from all methodologies for each characteristic. This allows for an exploration of the relationships and groupings of methods based on stability. The correlogram, representing the correlation coefficients, was generated using the metan package (Olivoto and Lúcio 2020). Principal component analysis (PCA) was performed on the ranking correlation matrix using the Factoextra package to further explore the relationships between method groups. All these analyses were implemented in R software version 4.2.0 (R Core Team 2022).

## Results

### Analysis of variance of the agronomic data

Based on the joint AMMI analysis of variance, the three sources of variation, genotypes, environments, and G × E interaction, were found to be significant (p < 0.001) for both traits (Table 4). The phenotypic expression of FRY and DMC varied among the evaluated genotypes, indicating the presence of selectable variation even with a small number of genotypes in the final evaluation phase. It is likely that the high variation in climatic factors (Fig. 1) contributed to the observed differences in the agronomic performance of cassava genotypes. The significance of the G × E suggests that genotypes responded differently to environmental stimuli based on their variable behavior across different evaluation environments. Therefore, quantifying and exploiting G × E through adaptability and stability parameters becomes necessary for more accurate recommendations of genotypes with greater agronomic interest.

**Table 4:** Analysis of variance by the additive main effects and multiplicative interaction (AMMI) method and sum of squares of the genotype × environment interaction for fresh root yield (FRY) and dry matter content (DMC), evaluated in 12 cassava genotypes in 12 environments

Source of variation	GL^1^	Sum of squares^2^	
		FRY	DMC
Genotypes (G)	11	8645.52***	1400.56***
Environments (E)	11	8780.89***	479.66***
Blocks/E	24	371.10***	24.45***
G × E	121	7279.50***	301.72***
PC1	21	2390.07***	115.66***
PC2	19	1506.66***	63.00***
Residue	264	1726.49	112.63
% SS due to the G	11	34.99	64.19
% SS due to the E	24	35.54	21.98
% SS due to the G × E	121	29.47	13.83
% G × E SS due to the PC1	21	32.83	38.33
% G × E SS due to the PC2	19	20.70	20.88
${\widehat{r}}_{\widehat{gg}}$		0.96	0.99
Overall average		26.14	36.22

^1^GL: Degree of freedom

***Significant at p < 0.001% probability, by F test

$${\widehat{r}}_{\widehat{gg}}$$: Selective accuracy

For FRY, the environment effect accounted for 35.54% of the total sum of squares (SQ), followed by the effect of genotypes and the G × E interaction, which contributed 34.99% and 29.46%, respectively (Table 4). The first two principal components (PC1 and PC2) of the AMMI biplot analysis explained 32.83% and 20.70% of the SQ of the G × E interaction. On the other hand, for DMC, the SQ of the effect of genotypes represented 64.18%, while the SQ of the environment and the G × E interaction accounted for 21.98% and 13.82%, respectively. For DMC, the two principal components captured 38.33% and 20.88% of the SQ.

The selective accuracy estimates (${\widehat{r}}_{\widehat{gg}}$) for both agronomic traits exceeded 95% (Table 4), indicating high experimental precision of the VCU tests, enabling reliable inferences about the average performance of the genotypes in the assessment environments. Estimates of genetic parameters exhibited wide variation for the two traits, indicating the potential for genetic gains. For FRY, genetic variance (${\sigma }_{g}^{2}$) contributed to 45% of the phenotypic variance (${\sigma }_{p}^{2}$), followed by G × E interaction variance (${\sigma }_{gxe}^{2}$) at 40.0% (Table 5). In the case of DMC, ${\sigma }_{g}^{2}$ and ${\sigma }_{gxe}^{2}$ accounted for 76.0% and 15.0% of the variance ${\sigma }_{p}^{2}$, respectively. Estimates of broad-sense heritability (${h}^{2}$) were relatively low for FRY (0.45) and high for DMC (0.75).

**Table 5:** Estimates of variance components and genetic parameters for fresh root yield (FRY) and root dry matter content (DMC), in 12 cassava genotypes evaluated in 12 environments

Parameters	FRY (t ha^−1^)	DMC (%)
${\sigma }_{g}^{2}$	20.16	3.46
${\sigma }_{gxe}^{2}$	17.87	0.68
${\sigma }_{e}^{2}$	6.54	0.42
${\sigma }_{p}^{2}$	44.57	4.56
${h}^{2}$	0.45	0.75
${CV}_{g}$	17.18	5.14
${CV}_{p}$	25.53	5.84
${CV}_{r}$	9.78	1.80
${CV}_{ratio}$	1.75	2.85
$P-G$	8.35	0.70
$*GA$	6.38	3.13
$GAM$	24.40	8.64

${\sigma }_{g}^{2}$—genetic variance, ${\sigma }_{gxe}^{2}$—genotype × environments interaction variance, ${\sigma }_{p}^{2}$ —phenotypic variance, ${\sigma }_{e}^{2}$ —residual variance,${h}^{2}$—broad sense heritability, ${CV}_{g}$ —genotypic coefficient of variation (%),${CV}_{p}$—phenotypic coefficient of variation (%), ${CV}_{r}$—residual coefficient of variation, ${CV}_{ratio}$ —relative coefficient of variation, $P-G$—difference between ${CV}_{p}$ and ${CV}_{g}$, $GA$—genetic gain (considering 15% selection intensity), GAM—genetic advance as a percentage of the mean

The high values of ${CV}_{g}$, ${CV}_{p}$ and ${CV}_{ratio}$ for both traits indicate that a significant portion of the observed variation is attributed to the effect of genotypes, particularly for DMC. The genetic advance estimates as a percentage of the average (GAM) of 24.40% (FRY) and 8.64% (DMC) suggest the potential for significant improvement, especially in fresh root productivity, through the selection of the best genotypes.

### Agronomic performance and comparison between the methods for G × E analysis

The range of root yield varied from 16.03 t ha^−1^ for the genotype IAC-90 to 33.57 t ha^−1^ for the genotype BR11-34–41. Other genotypes such as BR11-34–69 and BRS Formosa also exhibited high root productivity, with yields of 31.49 t ha^−1^ and 29.67 t ha^−1^, respectively. In general, the average root yield of the new clones was higher than that of the control varieties, with the six new clones averaging 27.89 t ha^−1^ compared to 24.38 t ha^−1^ for the controls.

Regarding DMC, among the five genotypes with high DMC, two new genotypes, BR11-24–156 and BR12-107–002, were among those with the highest average DMC (37.13% and 37.46%, respectively). On average, the six new clones exhibited a DMC of 35.43%, while the controls had an average of 37.01%. However, some genotypes, such as BR11-24–156 and BR12-107–002, surpassed the average DMC of the controls. The highest DMC average was observed in the BRS Novo Horizonte genotype (39.33%), which performed well in the NR-2019 and RA2-2019 environments, indicating a certain adaptability to these specific environments (Figure S1).

There was divergence in the classification of genotypes based on simultaneous selection for stability and agronomic performance. The genotype BR11-34–41 showed greater stability for FRY according to nine methods, ranking 1st based on the $HMGV$, ${Si}^{6}$, and three Annicchiarico indices. However, it showed high deviation based on the classification of multivariate methods such as $WAASB$, $ASV$, $DP$, and $Wi$, and non-parametric ${Si}^{1} and ASV$ (Table 6). Genotypes BR11-34–69, BR11-24–156, BR11-34–64, and BRS Novo Horizonte exhibited stability according to most of the methods used. On the other hand, genotypes IAC-90, BRS Mulatinha, and BRS Formosa were ranked as the most unstable for FRY.

**Table 6:** Phenotypic adjusted means and ranking (within parentheses) obtained by ordering stability values for fresh root yield (FRY), obtained from the evaluation of 12 cassava genotypes, in 12 environments

Genotypes	FRY	$DP$	WAASB	$ASV$	$GGE$	$Wi$	$Pi$	${Si}^{1}$	${Si}^{6}$	$HMGV$
BR11-24–156	24.64 g	5.41 (2)	0.50 (1)	0.89 (2)	0.32 (2)	64.67 (1)	76.62 (7)	0.06 (8)	4.12 (8)	23.4 (9)
Cigana Preta	22.14 h	7.10 (10)	1.17 (11)	1.57 (4)	− 0.29 (1)	235.16 (10)	111.58 (10)	0.11 (12)	6.97 (11)	20.2 (11)
Corrente	24.71 g	5.51 (4)	1.08 (10)	2.01 (6)	4.46 (7)	123.01 (3)	79.97 (9)	0.06 (8)	4.28 (9)	23.6 (8)
IAC-90	16.03i	6.37 (7)	0.73 (2)	2.61 (7)	− 5.76 (9)	200.80 (6)	224.17 (12)	0.03 (4)	7.27 (12)	14.0 (12)
BR11-34–41	33.57a	7.85 (12)	1.04 (8)	2.69 (8)	− 2.83 (4)	205.39 (7)	11.58 (1)	0.06 (8)	1.12 (1)	32.1 (1)
BR11-34–45	28.66d	7.63 (11)	0.75 (3)	3.65 (11)	− 9.35 (11)	213.30 (8)	38.21 (4)	0.03 (4)	3.77 (6)	27.2 (4)
BR11-34–64	26.53f	6.43 (8)	1.07 (9)	1.97 (5)	− 5.10 (8)	115.14 (2)	60.88 (6)	0.03 (4)	3.08 (3)	25.1 (7)
BR11-34–69	31.49b	6.98 (9)	1.55 (12)	3.03 (9)	− 3.80 (6)	227.14 (9)	19.36 (2)	0.08 (10)	2.69 (2)	30.2 (2)
BR12-107–002	22.50 h	5.13 (1)	0.86 (6)	0.76 (1)	− 2.85 (5)	161.39 (5)	113.67 (11)	0.09 (11)	5.18 (10)	21.6 (10)
BRS Formosa	29.67c	5.89 (5)	0.80 (4)	3.25 (10)	6.44 (10)	280.66 (11)	31.90 (3)	0.05 (6)	3.33 (4)	28.6 (3)
BRS Mulatinha	26.33f	5.49 (3)	0.81 (5)	5.72 (12)	17.03 (12)	450.70 (12)	77.02 (8)	0.02 (1)	3.69 (5)	25.4 (6)
BRS Novo Horizonte	27.40e	5.93 (6)	1.04 (7)	1.27 (3)	1.73 (3)	149.16 (4)	45.98 (5)	0.03 (4)	3.93 (7)	26.3 (5)
Genotypes	FRY	${S}_{di}^{2}$	RMSE	${R}^{2}$	$Bi$	$Di$	$li\left(g\right)$	$li\left(f\right)$	$li\left(d\right)$	${S}^{2}x$
BR11-24–156	24.64 g	4.27 (1)	2.31 (1)	0.80 (1)	0.03 (6)	6.45 (1)	86.9 (8)	89.0 (7)	85.4 (9)	6.0 (5)
Cigana Preta	22.14 h	20.76 (10)	4.37 (10)	0.59 (7)	0.15 (4)	22.94 (10)	70.9 (11)	78.9 (9)	65.3 (11)	8.11 (9)
Corrente	24.71 g	10.01 (3)	3.18 (3)	0.63 (5)	− 0.07 (9)	12.19 (3)	85.9 (9)	84.9 (8)	86.0 (8)	5.48 (3)
IAC-90	16.03i	17.90 (8)	4.09 (8)	0.55 (9)	0.00 (7)	20.08 (8)	48.0 (12)	58.2 (12)	42.7 (12)	4.0 (2)
BR11-34–41	33.57a	12.98 (6)	3.55 (6)	0.78 (2)	0.47 (1)	15.16 (6)	118.0 (1)	124.0 (1)	114.0 (1)	1.48 (1)
BR11-34–45	28.66d	15.72 (7)	3.86 (7)	0.72 (4)	0.38 (2)	17.9 (7)	99.0 (4)	99.7 (4)	98.0 (5)	7.75 (7)
BR11-34–64	26.53f	8.39 (2)	2.96 (2)	0.77 (3)	0.20 (3)	10.57 (2)	91.9 (6)	101.0 (3)	86.7 (7)	6.39 (6)
BR11-34–69	31.49b	20.11 (9)	4.30 (9)	0.58 (8)	0.13 (5)	22.29 (9)	109.0 (2)	105.0 (2)	110.0 (2)	8.45 (10)
BR12-107–002	22.50 h	12.57 (4)	3.50 (4)	0.49 (10)	− 0.24 (10)	14.75 (4)	76.7 (10)	73.3 (11)	78.5 (10)	5.73 (4)
BRS Formosa	29.67c	23.79 (11)	4.65 (11)	0.32 (11)	− 0.29 (11)	25.97 (11)	101.0 (3)	99.1 (5)	107.0 (3)	8.09 (8)
BRS Mulatinha	26.33f	29.35 (12)	5.12 (12)	0.05 (12)	− 0.75 (12)	31.53 (12)	88.6 (7)	75.5 (10)	99.6 (4)	9.2 (12)
BRS Novo Horizonte	27.40e	12.73 (5)	3.52 (5)	0.61 (6)	− 0.01 (8)	14.91 (5)	95.1 (5)	92.7 (6)	96.4 (6)	8.82 (11)

*Means followed by the same letter in the column belong to the same grouping by Scott-Knott test (p < 0.05); R = ranking; $DP$ = standard deviation; $WAASB$ weighted average of absolute scores; $ASV$= AMMI stability value; $GGE$= genotype scores PC2; $Wi$ = Ecovalence of Wricke's 1962; $Pi$ index of superiority over all environments of Lin e Binns; ${Si}^{1}$ and ${Si}^{6}$ = non-parametric indices of Nassar & Huehn (1987); $HMGV$ = harmonic mean of genetic values; $E\&R-{S}_{di}^{2}$ = regression variance, $E\&R-RMSE$ = mean square error ${E\&R-R}^{2}$ = the regression coefficient of determination (Eberhart; Russell, 1966); $P\&J-Bi$ = regression coefficient and $P\&J-Di$ = regression variance (Perkins & Jinks 1968); $li(g)$ genotypic confidence index with respect to all environments, $li(f$) favorable environments, $li(d)$ unfavorable environments (Annicchiarico 1992); ${S}^{2}x$= environmental variance

**Fig. 2:**
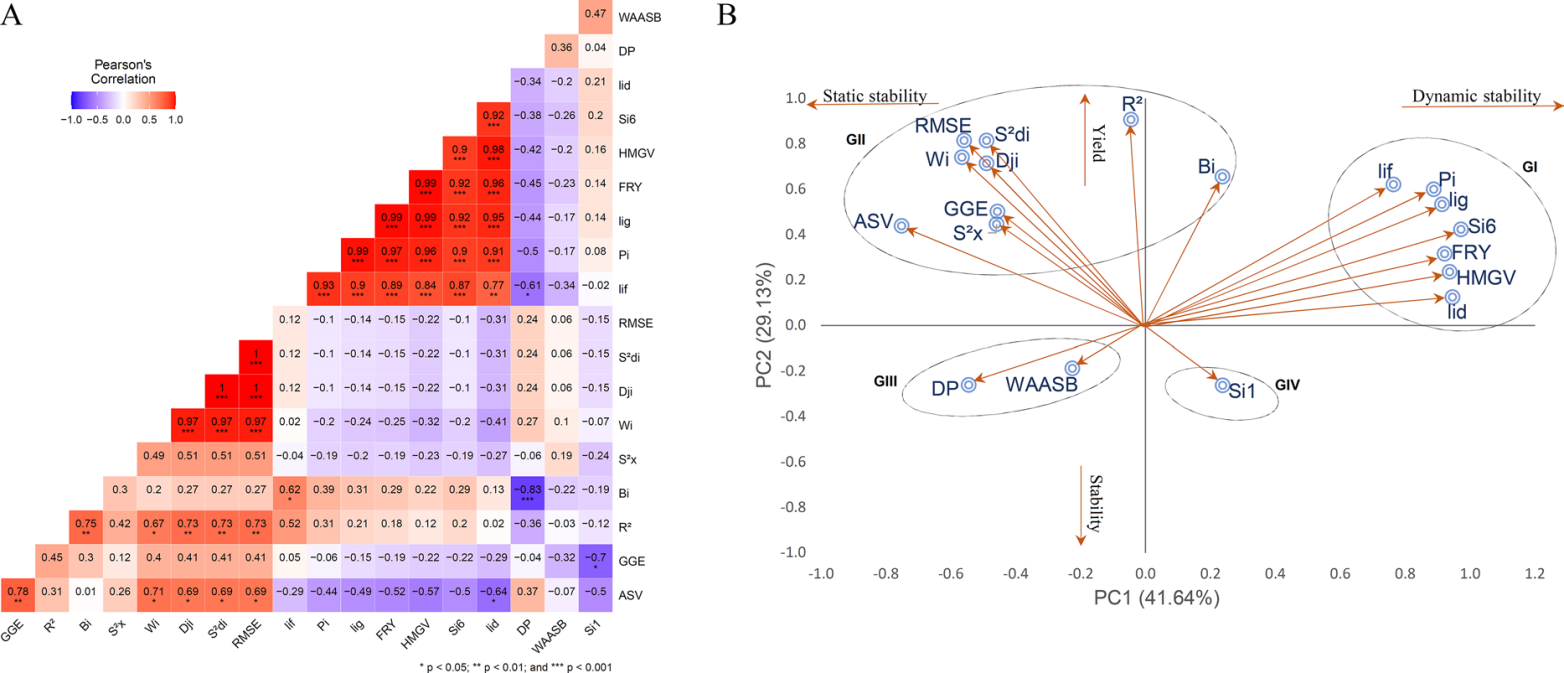
**A** Spearman's correlation coefficients and **B** biplot scatter plot of the first two principal components of adaptability and stability parameters for fresh root yield (FRY) evaluated in 12 cassava genotypes in multi-environments

For DMC, BRS Novo Horizonte was the most stable genotype according to thirteen methods, except for three univariate parametric methods ($DP$,$Wi$ and $Bi$) and one non-parametric method (${Si}^{1}$), as well as the GGE biplot analysis. Three other genotypes, Corrente, BR11-24–156, and BR11-34–69, also ranked high in terms of stability and adaptability. Conversely, genotypes IAC-90, BRS Mulatinha, and BR11-34–41 were the most unstable for DMC across the twelve evaluation environments, occupying positions between 9 and 12th in the ranking (Fig. 2).

### Correlations and clustering of the stability and adaptability methods

**Fig. 3:**
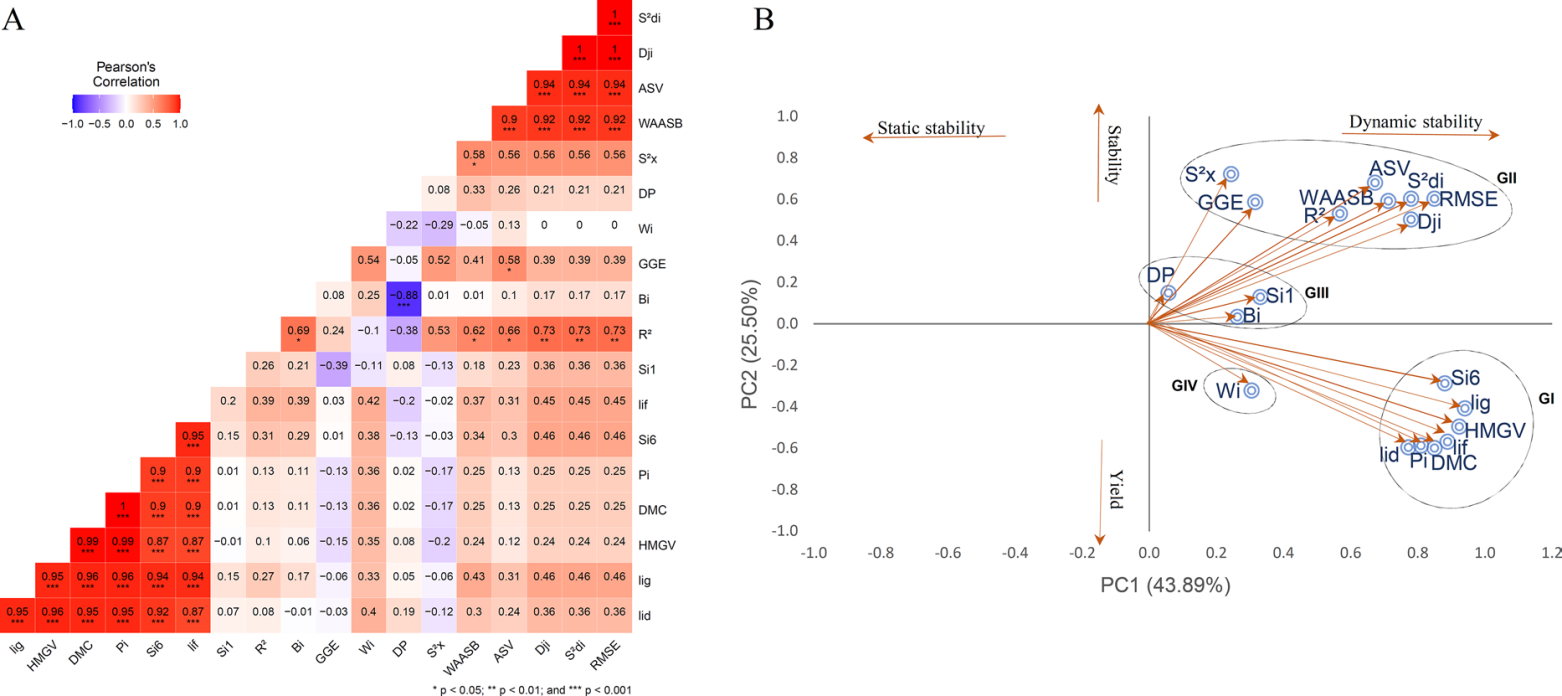
**A** Spearman correlation coefficients and **B** biplot scatter plot of the first two principal components of adaptability and stability parameters for root dry matter content (DMC) evaluated in 12 cassava genotypes in multi-environments

**Fig. 4:**
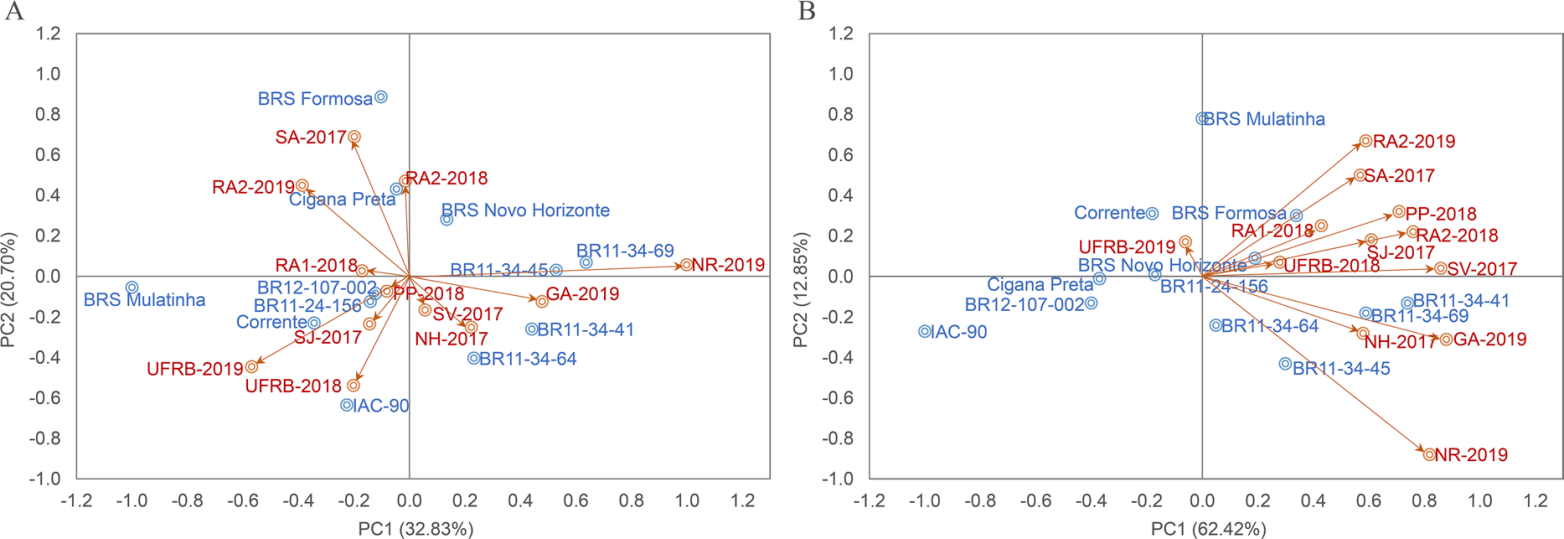
Biplot of adaptability and stability values based of additive main effects and multiplicative interaction – AMMI **A** and genotype main effects plus genotype x environment interaction effects – GGE, **B** methodologies for fresh root yield (FRY), from 12 cassava genotypes evaluated in multi-environments in the tropical humid region of Brazil

The Spearman correlations between adaptability and stability methods showed that approximately 23% and 24% of the correlations were significant (p-values < 0.01) and of high magnitude for FRY and DMC, respectively (Figs. 3A and 4A). These correlations were positive and different from zero, indicating associations between the methods. However, the magnitude and direction of the correlations varied, ranging from − 0.83 to 1.0 for FRY and − 0.88 to 1.0 for DMC, indicating that most methods were complementary but with some disagreement in the classification of genotypes in terms of adaptability and stability.

For FRY, high correlations (0.99, 0.98, and 0.84) were observed between the Annicchiarico parameters $li(g)$, $li(d)$, $li\left(f\right)$ and the harmonic mean $HMGV$ (Fig. 2). There were also high correlations (r ≥ 0.80) between these three parameters and environmental variance (${Si}^{6}$) as well as maximum correlations between RMSE and ${S}^{2}di$ and $Dji$. Approximately 65.0% of the methods showed low correlations (− 0.40 ≤ r ≤ 0.40) for both characteristics, suggesting their use as complementary methods of analysis. Only 1.5% of the methods exhibited a significant negative correlation of high magnitude, such as $Bi$ vs $DP$ (rs =  − 0.83***), which can be used as a complementary approach for selecting low-yield genotypes to be discarded in the breeding program.

For DMC, correlations greater than 0.90 were observed between Huehn's parameter (${Si}^{6}$) and Annicchiarico's parameters ($li(g)$, $li(f)$ and $li(d)$) as well as with the ranking based on the mean of the trait (Fig. 3). These correlations indicate that these four parameters are associated with high stability and higher DMC contents. The $HMGV$ method also showed a strong positive association with DMC (0.99), indicating a strong relationship between agronomic performance and DMC.

Multivariate statistical methods ($ASV$ and $GGE$) showed positive correlations among themselves, ranging from 0.58 for DMC to 0.78 for FRY. This indicates that these approaches have the same correlation direction but may vary depending on the specific trait being analyzed, suggesting their complementary use. On the other hand, the two linear regression-based methods ($Dji$ and $Bi$) showed low associations for both FRY and DMC, suggesting that the environmental variance can influence the response pattern of these methods due to changes in genotype performance across different environments.

The first two principal components (PCs) explained 71.78% of the phenotypic variation for FRY and 67.47% for DMC (Fig. 2B and 3B), with both characteristics being divided into four groups to allow for classification based on the static (biological) and dynamic (agronomic) concepts of stability. For both FRY and DMC, Group I comprised six methods ($li(g)$, $li(f)$, $li(d)$, ${Si}^{6}$, $Pi$ and $HMGV$) with significant, positive correlations (superior to 0.77 for FRY and 0.87 for DMC) among them. The phenotypic data for FRY and DMC were also allocated to this group, indicating a strong association between these characteristics and the stability parameters of Group I, and thus, dynamic stability.

Group II for FRY consisted of nine methods (${R}^{2}$, $Dji$, $Bi$, ${S}^{2}di$, $GGE$, $ASV$, $RMSE Wi$ and ${S}^{2}x$), including a negative and significant correlation with FRY (r ≥  − 0.40). Although the ${R}^{2}$ and $Bi$ methods were included in this group, their FRY values were of intermediate magnitude, characterizing the group by greater static stability, where genotype performance is relatively constant across most environments regardless of the magnitude of the evaluated characteristic. Group III comprised only the $DP$ and $WAASB$ methods, while Group IV consisted of the ${Si}^{1}$ method, which showed a low correlation with FRY, indicating static stability.

For DMC, Group II can be characterized by the dynamic concept with methods such as ${R}^{2}$, $Dji$, $WAASB$, ${S}^{2}di$, $GGE$, $ASV$, $RMSE$ e ${S}^{2}x$, as they were associated with the same direction of the response variable. Groups III and IV included three and one stability methods, respectively, with moderate associations with DMC.

In general, methods based on variance estimates ($$RMSE$$, $${R}^{2}, {S}^{2}di$$, $$Dji$$ and $${S}^{2}x$$) tended to classify genotypes similarly, as did the $${Si}^{6}$$ and $$HMGV$$ parameters, which simultaneously select for stability and performance. For FRY, there were significant and positive correlations (r ≥ 0.87***) between univariate parametric and non-parametric methods $$(lig/{Si}^{6}$$, $$lif/{Si}^{6}, lid/{Si}^{6}$$ and $$Pi/{Si}^{6}$$); four correlations between univariate and multivariate parametric methods (r ≥ 0.69*) ($$Wi/ASV$$; $$Dji/ASV$$, $${S}^{2}di/ASV)$$ and $$RMSE/ASV$$); and four correlations between parametric univariate and mixed models (r ≥ 0.84***) ($$lig/HMGV$$; $$lif/HMGV$$; $$lid/HMGV$$ and $$Pi/HMGV$$). One correlation between the mixed model and non-parametric method was significant and negative (r =  − 0.70*) between ($$MHVG$$ and $${Si}^{6}$$. For DMC, the results were similar, except for the correlations between univariate and multivariate parametric methods, which were not significant, indicating random classification similarity among some genotypes. There was also no significant positive correlation between the mixed and multivariate models for both traits.

The results obtained for DMC showed similar trends, with the exception of the four correlations observed between univariate and multivariate parametric methods (r ≥ 0.66*) such as ($${R}^{2}/ASV$$; $$Dji/ASV$$, $${S}^{2}di/ASV)$$ and $$RMSE/ASV$$. On the other hand, the correlations between nonparametric and multivariate methods were not found to be significant, indicating that certain classifications of genotypes were randomly assigned. Furthermore, there was no positive and significant correlation observed between the mixed and multivariate model for the two characteristics.

Among the various methods employed, ${S}^{2}di/Dji,RMSE/Dji$ and $RMSE/ASV$ exhibited the highest correlation values (r ~ 1.0) for FRY and DMC. Consequently, some of these methods can be disregarded in the analysis. However, it is worth noting that the complementarity between certain methods can aid in the selection of the most suitable genotypes, particularly those based on multivariate approaches and mixed models, which consider simultaneous selection for stability and yield.

### Stability analysis: ${\varvec{A}}{\varvec{M}}{\varvec{M}}{\varvec{I}}$ x ${\varvec{G}}{\varvec{G}}{\varvec{E}}$ x ${\varvec{W}}{\varvec{A}}{\varvec{A}}{\varvec{S}}{\varvec{B}}$ x ${\varvec{H}}{\varvec{M}}{\varvec{G}}{\varvec{V}}$

**Fig. 5:**
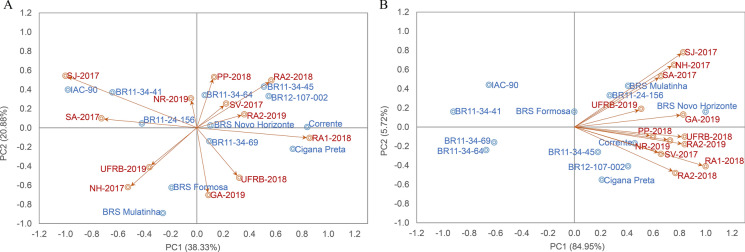
Biplot of adaptability and stability values based of additive main effects and multiplicative interaction – AMMI **A** and genotype main effects plus genotype x environment interaction effects – GGE, **B** methodologies for dry matter content (DMC), from 12 cassava genotypes evaluated in multi-environments in the tropical humid region of Brazil

**Fig. 6:**
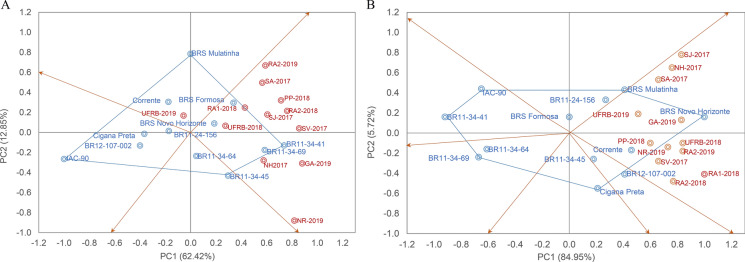
Biplots of which-won-where, genotype main effects plus genotype x environment interaction effects (GGE) for: (**A**) fresh root yield (FRY) and; (**B**) dry matter content (DMC), obtained from from 12 cassava genotypes evaluated in multi-environments in the tropical humid region of Brazil

Utilizing multivariate models, such as the GGE biplot methodology, enables a more efficient and reliable exploration of the genotype-by-environment (G × E) interaction in multi-environment trials. The GGE analysis explained a greater proportion of the variation in both FRY and DMC compared to the AMMI analysis. The first two principal components of the GGE analysis captured 21.74% more SQG × E variation for FRY and 31.45% more for DMC compared to the AMMI analysis (Figs. 5 and 6). The AMMI analysis had double the residual variation (42.20%) compared to the GGE analysis (21.22%), indicating a greater influence of factors without biological explanation in the AMMI model. The environmental factor represented a higher proportion of the sum of squares for FRY, while the genotypic factor was higher for DMC.

In the AMMI methodology, genotypes close to the origin of the axes are less sensitive to the G × E interaction, while those further away from the origin are more sensitive and influenced by the environment. For FRY, three genotypes (BR12-107–002, BR11-24–156, and BRS Corrente) showed a low contribution to the G × E interaction (Fig. 4). The BR11-24–156 genotype ranked well according to several stability methods, including WAASB, with a median FRY (Table 6). For DMC, the most stable genotypes were BR11-34–69 and BRS Novo Horizonte, with the latter also showing wide adaptation due to its proximity to the center of the biplot and high FRY according to the HMGV and WAASB methods (Fig. 5).

Based on the GGE method, genotypes and environments located to the right of the biplot (PC1 axis) tend to have higher yields. Therefore, the BR11-34–69 and BR11-34–41 genotypes showed high performance with stability, indicated by their low angle with PC2. BR12-107–002 and BR11-24–156, along with the controls BRS Novo Horizonte and Cigana Preta, exhibited greater overall stability for FRY (Fig. 4). For DMC, clones BR11-34–41, BR11-34–69, BR11-34–45, and BR11-34–64 showed stability, while the Corrente controls displayed general stability and BRS Novo Horizonte showed high performance and stability (Fig. 5).

Considering the environmental analysis using the AMMI method, three environments (PP-2018, RA1-2018, and NR-2019) exhibited edaphoclimatic conditions that allowed for greater stability in FRY, and three environments (SA-2017, RA1-2018, and RA2-2019) did so for DMC. Only the RA1-2018 environment was common for both traits. On the other hand, the contribution of the G × E interaction captured by the GGE methodology varied greatly depending on the trait. The UFRB-2018, UFRB-2019, and SV-2017 environments facilitated greater stability for FRY, while four other environments (NR-2019, GA-2019, RA2-2019, and PP-2018) had a low influence on the phenotypic expression of DMC (Figs. 5 and 6).

The WAASB analysis incorporates both the AMMI technique and best linear unbiased predictions (BLUPs) while utilizing all the axes of the principal components (PCs). In the WAASB analysis for FRY, two quadrants are particularly important: i) upper right quadrant that includes the BR11-34–41 and BR11-34–69 genotypes with high yield but some instability. Additionally, the NR-2019 environment demonstrates a high ability to discriminate between genotypes; ii) lower right quadrant where the BR-34–64 and BRS Novo Horizonte genotypes are allocated. These genotypes exhibit above-average productivity and low WAASB values, indicating wide adaptation and stability, albeit with moderate yield (7th and 5th for HMGV, respectively). For DMC, BRS Novo Horizonte, located in the lower right quadrant, demonstrates high performance and stability. This is evident from Table 6 and Figure S2. By conducting a joint analysis of the GGE and AMMI methods, breeders can increase their confidence in recommending new genotypes. This integrated approach provides a comprehensive understanding of genotype performance and stability across multiple environments, enabling better decision-making in genotype selection and recommendation.

### Specific adaptability

Overall, the AMMI analysis revealed a low correlation between the environments, primarily due to the significant dispersion of the 12 environments in the biplots for the two agronomic traits. However, this increased dispersion actually facilitates the identification of genotypes that are better suited to specific conditions, thereby maximizing gains in different environments. Notably, specific adaptations for FRY were observed in genotypes IAC-90 (UFRB-2018), Cigana Preta (RA2-2018), and BRS Formosa (SA-2017), followed by genotypes BR11-24–156 and 2012–107-002 in environments PP-2018, RA1-2018, and SJ-2017 (Fig. 4). Regarding DMC, genotypes BR12-107–002 and BR11-34–45 exhibited high adaptability in the RA2-2018 environment, while BR11-34–41 and IAC-90 performed well in the SJ-2017 environment. Genotypes BR11-34–64 excelled in the NR-2019 environments, and PP-2018 and BR11-34–69 showed adaptability in the UFRB-2018 environment (Fig. 5).

In the GGE analysis, the angulation between the eigenvectors of genotypes and environments offers valuable insights into specific adaptabilities. According to Yan (2016), an angle smaller than 90° in the GGE biplot indicates that the performance of the corresponding genotype surpasses the average, exhibiting a high positive correlation. Conversely, angles above 90° suggest below-average performance with a negative correlation, while angles close to 90° indicate performance similar to the mean. Regarding FRY, genotypes 2011–34-41 and 2011–34-69 demonstrated specific adaptations to the NH-2017 and GA-2019 environments, respectively, followed by the control genotype BRS Formosa in the RA1-2018 environment. The control genotype BRS Novo Horizonte exhibited an angle of less than 90° in all environments for both traits, highlighting its high adaptive potential, although it achieved the best FRY and DMC in the UFRB-2018 and GA-2019 environments, respectively. Among the six new genotypes evaluated with potential for cultivation recommendation, three (2011–34-41, 2011–34-64, and 2011–34-69) and the control genotype IAC-90 presented DMC values below the average for all evaluated environments (Figs. 5 and 6).

The interrelationship between environments can also be explored through the biplot. For FRY, the NR-2019 environment exhibited a positive correlation with all other environments, except UFRB-2019. As for the DMC variable, all environments displayed some degree of correlation, with angles smaller than 90° in all analyzed environments. Overall, the environments demonstrated similar ability to discriminate among the evaluated genotypes. In the WAASB method, the first quadrant showcases environments with a high discrimination capacity, indicating the need to explore specific adaptations. For instance, IAC-90 exhibited promising performance in the RA2-2019 and SA-2017 environments for FRY, while IAC-90 and BR11-34–41 displayed potential in the SJ-2017 and NH-2017 environments for DMC (Figure S2).

### Mega-environments identification

The GGE analysis provides a valuable tool for exploring the "which-won-where" pattern, which involves forming a polygon connecting genotypes located farthest from the origin of the biplot. This approach emphasizes the correlation between environments and genotype performance, leading to the identification of mega-environments. In the case of the FRY variable, the first two principal components explained 62.42% and 12.85% of the variance, respectively (Fig. 6). The biplot was divided into four sectors by the rays, with only two of them representing mega-environments. Group 1 consisted of nine environments, including UFRB-2018, PP-2018, and NR-2019, with the genotype BR11-34–41 at its apex, indicating the highest FRY. This was followed by genotypes BR11-34–69 and BRS Novo Horizonte. Group 2 comprised two environments, namely UFRB-2019 and RA2-2019, with BRS Mulatinha at the apex. These environments exhibited similar characteristics, leading to their grouping. However, the IAC-90 genotype displayed below-average yield.

The equality line (represented by red arrows) divides the biplot into sectors and represents equal values between two genotypes in a given environment. For example, IAC-90 and BRS Mulatinha showed equal values for FRY in hypothetical columns within this row. Regarding DMC, the genotypes BR11-34–41, BR11-34–69, BR12-107–002, BRS Novo Horizonte, BRS Mulatinha, IAC-90, and Cigana Preta formed the vertices, with BRS Novo Horizonte being the main representative of the vertex, indicating the highest DMC across all environments.

Although the polygon consists of six sectors, only one mega-environment was formed due to smaller environmental influence on this variable. Additionally, the variation within the environment was greater than between groups. The equality line indicated equal values between IAC-90 and the BR11-34–41 genotype in some hypothetical environments among the evaluated assays.

The environments were grouped based on their projection and vector length in the biplot graph, which resulted from the effects of representativeness and discrimination. The discrimination capacity of an environment was determined by the length of its vector in the biplot, while representativeness was measured by the cosine of the angle between the test environment and an ideal environment (Yan and Holland 2010).

The environments in Group 1 exhibited a high representativeness capacity, indicating consistent means with low standard deviation. For instance, the UFRB-2018 and UFRB-2019 environments for FRY, as well as the PP-2018 and NR-2019 environments for DMC, displayed short vectors, indicating stability in performance over the years. While these environments may not effectively discriminate phenotypic variations among genotypes, they can be considered representative and stable. In contrast, the environments in Group 2 (GA-2019 and SV-2017 for FRY, GA-2019 and UFRB-2018 for DMC) had long vectors and small angles with the AEC axis, making them more discriminating and suitable for selecting highly adapted genotypes with high yield. These environments are more informative and can effectively detect differences between genotypes. The positive correlations between the environments in Groups 1 and 2, indicated by the angles smaller than 90°, suggest that genotypes exhibit similar responses in these environments. Utilizing these environments alternately allows for minimal loss of information.

The environments in Group 3 have long vectors and large angles, indicating high discriminative capacity. These environments are informative for detecting differences between genotypes due to their high standard deviation. However, they are strongly influenced by G × E interactions and may not be suitable for selecting superior genotypes. Nonetheless, they can be useful for better classifying genotypes based on their instability or identifying genotypes with specific adaptations to certain environments. Examples include NR-2019 and RA2-2019 for FRY, and NH-2017, SJ-2017, and RA1-2018 for DMC. These environments are not correlated, as indicated by the angles larger than 90° (Yan and Tinker 2006).

## Discussion

### Genetic parameters in cassava VCUs trials

Cassava, being an allogamous species with a high cross-pollination rate, exhibits a significant degree of heterozygosity in its genome (Halsey et al. 2008). This characteristic leads to substantial variability between and within progenies, particularly during the initial and intermediate stages of selection (Ceballos et al. 2012). This genetic variability, combined with environmental factors, contributes to the significant genotypic effects (> 35% for FRY and > 64% for DMC) and G × E interactions (29% for FRY and 13% for DMC) observed in the phenotypic expression. Consequently, conducting MET is crucial to unravel the G × E interactions, explore predictable responses to different environments, and assess stability and adaptability, ultimately advancing breeding efforts.

Previous studies have reported substantial variation in the G × E interaction for FRY and DMC, with variances of 43.80% and 49.08%, respectively (Adjebeng-Danquah et al. 2017; Jiwuba et al. 2020). The genetic control of these traits helps explain these findings. FRY is influenced by numerous genes with a quantitative and polygenic nature, making it highly susceptible to environmental influences (Ssemakula et al. 2007; Wolfe et al. 2016). As a result, FRY exhibits moderate broad-sense heritability (${h}^{2}$> 0.45). On the other hand, DMC is governed by a few genes, leading to low variation between genotypes and high heritability ${(h}^{2}$ > 0.75) due to reduced environmental influence (Ceballos et al. 2015).

In VCU trials, a high genetic variance plays a crucial role in selecting genotypes with diverse response patterns across different growing environments, enabling subsequent planting recommendations. The joint analysis of variance confirmed the significance (p < 0.01) of both environmental effects and the G × E interaction. For DMC, the genetic variation was predominant over the environmental variation, while the opposite situation was observed for FRY. These findings align with the results reported by Adjebeng-Danquah et al. (2017), who analyzed the stability and performance of cassava genotypes in multi-environment trials in Ghana, Africa. In their study, they reported that the genetic, environmental, and G × E interaction components accounted for approximately 32.77%, 37.55%, and 29.68% of the phenotypic variation for FRY, and 54.10%, 9.78%, and 36.10% for DMC, respectively.

Based on these results, different criteria should be applied in the development of new cassava varieties. For FRY, there are indications of a possible dominance of non-additive genetic action with low heritability ${(h}^{2}$), suggesting the application of methods based on recurrent phenotypic selection or the exploration of heterotic effects through crossing different genetic pools (Tumuhimbise et al. 2014). On the other hand, the higher heritability estimates for DMC and the potential predominance of additive gene effects suggest the use of simple phenotypic selection methods with increased selection intensity and a smaller number of environments, given their higher predictive capacity (Wolfe et al. 2016; Andrade et al. 2019). Therefore, heritability estimates provide valuable guidance to breeders in determining the most appropriate breeding methods, selection strategies, and experimental arrangements to minimize experimental errors and maximize genetic gains in each selection cycle (Oliveira et al. 2015).

### Yield potential of new cassava genotypes

Despite having contrasting genetic controls, our findings suggest that there is potential for improvement in both traits. The genetic advance as a percentage of the mean (*GAM*) provides a measure of the actual progress, and despite FRY demonstrating moderate heritability, significant gains can still be achieved due to the substantial genetic variation present in the germplasm, despite the adverse environmental effects when compared to DMC. The selection of the best genotypes in relation to the control varieties has resulted in noteworthy advancements in genetic improvement, even in the face of the significant G × E. Out of the six new clones evaluated, five (BR11-24–156, BR11-34–41, BR11-34–64, BR11-34–69, and BR11-34–45) exhibited FRY values exceeding 24.6 t ha^−1^. Additionally, two highly productive clones (BR11-24–156 and BR11-34–45), along with the BR12-107–002 genotype, displayed high DMC (> 36.69%). While the BRS Novo Horizonte genotype had the highest DMC, the BR11-34–41 and BR11-34–69 genotypes exhibited high FRY but relatively lower DMC values of 33.45% and 33.68% respectively. Consequently, it is crucial to prioritize the introgression of alleles associated with DMC in new cassava clones, aiming to achieve a more balanced combination of different characteristics within a single genotype (Table 6).

In the study area, cassava holds significant economic and social importance, encompassing a cultivation area of approximately 389,000 hectares and a gross production value of around R$1.12 billion. The region ranks as the third-largest cassava producing area in Brazil. However, the root yield is considerably low, standing at 9.80 t ha^−1^, even when compared to the national average of 14.9 t ha^−1^ (IBGE, 2021). On the other hand, current studies in Northeast Brazil indicate the potential for genetic advances of approximately 14% with the new genotypes, which translates to a substantial increase in root yield, around 3.49 t ha^−1^, compared to the average of the region's varieties. With an average price of R$567.60 per ton (adjusted for inflation over the past 10 years), cultivating these new genotypes could yield an additional value of approximately R$1,980.00 per hectare. Similarly, although the *GAM* for DMC was relatively lower (approximately 8.6%), it is possible to achieve additional profitability in cassava starch production, amounting to R$2,660.00 per hectare. This calculation considers the average deflated price of starch over the last 10 years, which stood at R$3,803.67 per ton (CEPEA et al. 2022), and a starch productivity of 8.58 and 7.88 t ha^−1^ for the improved clones and local varieties analyzed in this study respectively (Table 7).

**Table 7:** Phenotypic adjusted means and ranking (within parentheses) obtained by ordering stability values for dry matter content in roots (DMC), obtained from the evaluation of 12 cassava genotypes, in 12 environments

Genotypes	DMC	$DP$	WAASB	$ASV$	$GGE$	$Wi$	$Pi$	${Si}^{1}$	${Si}^{6}$	$HMGV$
BR11-34–45	36.69c	1.77 (11)	0.59 (11)	1.49 (7)	− 1.08 (7)	14.10 (3)	3.84 (7)	0.02 (2)	2.56 (5)	36.6 (7)
Cigana Preta	36.80c	1.77 (12)	0.56 (9)	1.96 (11)	− 1.84 (12)	31.60 (10)	3.83 (6)	0.06 (7)	2.79 (6)	36.7 (6)
BR11-34–64	34.14d	1.49 (8)	0.25 (3)	0.52 (3)	− 0.98 (5)	30.35 (8)	13.93 (10)	0.05 (5)	4.35 (9)	34.1 (10)
BR12-107–002	37.46b	1.56 (10)	0.49 (7)	1.54 (8)	− 1.29 (8)	15.71 (4)	2.25 (3)	0.03 (4)	1.84 (4)	37.4 (4)
Corrente	37.90b	1.43 (7)	0.36 (5)	1.17 (6)	− 0.72 (4)	10.01 (2)	1.34 (2)	0.06 (7)	1.16 (2)	37.9 (2)
BRS Novo Horizonte	39.33a	1.25 (6)	0.18 (1)	0.27 (1)	0.67 (3)	25.26 (7)	0.01 (1)	0.09 (11)	0.28 (1)	39.3 (1)
BR11-34–41	33.45e	1.50 (9)	0.57 (10)	1.78 (10)	0.66 (2)	22.20 (6)	18.10 (12)	0.11 (12)	4.92 (12)	33.4 (12)
BR11-34–69	33.68e	1.21 (3)	0.23 (2)	0.32 (2)	− 1.06 (6)	41.27 (11)	16.55 (11)	0.02 (2)	4.89 (11)	33.6 (11)
BRS Formosa	36.23c	1.23 (5)	0.41 (6)	1.03 (4)	0.65 (1)	6.59 (1)	5.41 (8)	0.08 (9)	3.05 (7)	36.2 (8)
BR11-24–156	37.13b	1.10 (2)	0.35 (4)	1.11 (5)	1.39 (9)	30.95 (9)	2.78 (5)	0.00 (1)	1.75 (3)	37.1 (5)
BRS Mulatinha	37.46b	1.09 (1)	0.55 (8)	1.60 (9)	1.79 (10)	18.52 (5)	2.33 (4)	0.06 (7)	3.21 (8)	37.4 (3)
IA-C90	34.35d	1.22 (4)	0.77 (12)	2.66 (12)	1.81 (11)	55.14 (12)	13.65 (9)	0.08 (9)	4.80 (10)	34.3 (9)
Genotypes	DMC	${S}_{di}^{2}$	RMSE	${R}^{2}$	$Bi$	$Di$	$li\left(g\right)$	$li\left(f\right)$	$li\left(d\right)$	${S}^{2}x$
BR11-34–45	36.69c	0.63 (7)	0.80 (7)	0.77 (4)	0.42 (1)	0.78 (7)	96.97 (7)	99.11 (5)	96.58 (6)	3.45 (9)
Cigana Preta	36.80c	0.66 (9)	0.82 (9)	0.76 (5)	0.41 (2)	0.81 (9)	97.12 (6)	98.31 (6)	95.38 (8)	5.27 (11)
BR11-34–64	34.14d	0.31 (4)	0.61 (4)	0.81 (2)	0.22 (3)	0.46 (4)	90.98 (9)	91.76 (9)	89.81 (10)	1.45 (4)
BR12-107–002	37.46b	0.65 (8)	0.81 (8)	0.70 (7)	0.19 (4)	0.79 (8)	99.45 (4)	101.42 (2)	97.31 (5)	3.36 (8)
Corrente	37.90b	0.45 (5)	0.70 (5)	0.73 (6)	0.11 (5)	0.60 (5)	101.28 (2)	100.83 (3)	102.24 (2)	2.27 (5)
BRS Novo Horizonte	39.33a	0.07 (1)	0.42 (1)	0.87 (1)	0.06 (6)	0.21 (1)	106.57 (1)	107.32 (1)	105.78 (1)	0.18 (1)
BR11-34–41	33.45e	0.91 (11)	0.93 (11)	0.57 (9)	0.03 (7)	1.05 (11)	87.85 (12)	88.27 (11)	86.83 (12)	1.45 (3)
BR11-34–69	33.68e	0.19 (2)	0.52 (2)	0.79 (3)	− 0.02 (8)	0.33 (2)	90.42 (10)	91.28 (10)	89.56 (11)	1.18 (2)
BRS Formosa	36.23c	0.56 (6)	0.76 (6)	0.57 (10)	− 0.15 (9)	0.71 (6)	96.26 (8)	96.99 (8)	96.35 (7)	4.36 (10)
BR11-24–156	37.13b	0.28 (3)	0.59 (3)	0.67 (8)	− 0.17 (10)	0.43 (3)	99.47 (3)	99.36 (4)	99.33 (3)	2.55 (6)
BRS Mulatinha	37.45b	0.86 (10)	0.91 (10)	0.22 (11)	− 0.53 (11)	1.01 (10)	98.27 (5)	97.98 (7)	98.85 (4)	6.45 (12)
IA-C90	34.35d	1.25 (12)	1.08 (12)	0.14 (12)	− 0.57 (12)	1.40 (12)	89.08 (11)	87.47 (12)	92.70 (9)	3.09 (7)

*Means followed by the same letter in the column belong to the same grouping by Scott-Knott test (*p* < 0.05)

R = ranking; $DP$ = standard deviation; $WAASB$ weighted average of absolute scores; $ASV$= AMMI stability value; $GGE$= genotype scores PC2; $Wi$ = Ecovalence of Wricke's 1962; $Pi$ index of superiority over all environments of Lin e Binns; ${Si}^{1}$ and ${Si}^{6}$ = non-parametric indices of Nassar & Huehn (1987); $HMGV$ = harmonic mean of genetic values; $E\&R-{S}_{di}^{2}$ = regression variance, $E\&R-RMSE$ = mean square error ${E\&R-R}^{2}$ = the regression coefficient of determination (Eberhart; Russell, 1966); $P\&J-Bi$ = regression coefficient and $P\&J-Di$ = regression variance (Perkins & Jinks 1968); $li(g)$ genotypic confidence index with respect to all environments, $li(f$) favorable environments, $li(d)$ unfavorable environments (Annicchiarico 1992); ${S}^{2}x$= environmental variance

Cassava exhibits wide phenotypic variation, even through asexual propagation, due to factors such as the low uniformity of plant material and the diverse edaphoclimatic conditions. For instance, the precipitation in this study ranged from 1198.3 mm (UFRB-2018) to 689.43 mm (RA2-2019), demonstrating the significant G × E (Fig. 1). The substantial variation observed between evaluated environments, even within the same region (State of Bahia, Brazil), tends to elicit different responses from the clones during their physiological and productive cycles. Cassava, requiring approximately 400 mm of rain in the initial months of planting (1 to 5 MAP) and a temperature around 25ºC, displays remarkable adaptability and resilience in the face of climate variations, as evidenced by the highly variable meteorological data across years and evaluation periods (El-Sharkawy 2007, 2012; Tironi et al. 2019; Dwamena et al. 2022).

The extent of environmental influence can be quantified by analyzing the amplitude and the ratio between the genotypic variation coefficient (${CV}_{g}$) and the phenotypic variation coefficient (${CV}_{p}$). Values below 1 indicate an unfavorable situation for selection (Akinwale et al. 2011). Both FRY and DMC displayed higher ${CV}_{p}$ values compared to ${CV}_{g}$, indicating a significant environmental effect and demonstrating that the variability is not solely of genetic origin. However, this effect was more pronounced for FRY, with a ${CV}_{g}$ of 17.18 and ${CV}_{p}$ of 25.53, while it was less prominent for DMC, with a ${CV}_{g}$ of 5.14 and ${CV}_{p}$ of 5.84. Similar findings of lower ${CV}_{g}$ values for CMD have been reported by other authors. For instance, Peprah et al. (2020) observed significance for the genotypic effect on yield variables of roots and DMC, with ${CV}_{g}$ and ${CV}_{p}$ values of 15.58 and 17.63 for FRY, and 8.0 and 8.5 for DMC, respectively. In another study, authors reported a greater magnitude of ${CV}_{p}$ for FRY and DMC (Kundy et al. 2015; Ewa et al. 2017).

In summary, situations like these make it challenging to directly select the best genotypes when aiming for genetic progress in breeding programs. Therefore, strategies for obtaining genotypes with high performance and stability for each trait must differ. Alternatively, it is suggested to select genotypes for specific environments as a means to achieve more promising results, in terms of performance and stability, using various methods for both FRY and DMC.

### Comparison between the stability and adaptability methods

When comparing different methods for studying the G × E interaction in cassava, it was found that the methods of phenotypic stability analysis showed greater concordance in the classification of genotypes when there was a higher explanation of genetic variance over the environmental effect. For example, in the case of FRY, 47% of the methods ranked the best genotype between 1st and 2nd place, while for DMC, this percentage was higher at 72%. This suggests that when the environmental effect is relatively low, stability methods tend to be more consistent in identifying the best-performing genotypes.

Stability methods aim to explore and explain the patterns and biological evidence present in genotypes and environments. In the clustering analysis using principal component analysis (PCA), it was observed that DMC showed positive loadings along PC1 for all models, indicating a more consistent pattern in the ranking order of genotypes. On the other hand, FRY showed loadings of different directions, suggesting more discrepancies in the ranking order of genotypes among the stability methods. This implies that combining multiple stability methods could be a more effective approach to explore the G × E interaction in cassava breeding (Van Eeuwijk et al. 2016).

It is worth noting that recent publications have shown a strong trend towards using mixed models to explore the G × E interaction in breeding programs. However, in the PCA grouping, a clear distinction was observed for group I, where the methods most correlated with FRY and DMC were allocated. These methods are associated with the concept of agronomic stability, wherein a genotype is considered stable if its response is consistent with changes in the environment. Genotypes in group I exhibited a high capacity to respond to environmental stimuli, making them suitable for cultivation in commercial areas with a high technological level.

In the field of agronomy, stability refers to the ability of genotypes to respond differently to environmental conditions, enabling the selection of the best-performing genotype (Becker and Leon 1988). For instance, genotypes BR11-34–41 and BR11-34–69 exemplify this concept for FRY, while BRS Novo Horizonte and Corrente demonstrate agronomic stability for DMC. Therefore, these genotypes are recommended for planting in both favorable and unfavorable environments, as well as unpredictable conditions, as they exhibit positive responses to environmental improvements. This is supported by the $li(g)$ index, which measures genotypic confidence across all environments, $li(f$) for favorable environments, and $li(d)$ for unfavorable environments (Annicchiarico 1992). Stable genotypes with wide adaptation, assigned to group I, exhibit high agronomic performance in both favorable and unfavorable environments, while adaptable genotypes tend to excel in specific environments (Adetoro et al. 2021).

On the other hand, groups II and III, which exhibit weak or no association with the analyzed traits, are classified as statically stable (for FRY) and dynamically stable (although weakly associated) for DMC. The variance of the genotypes significantly influences the stability methods. For instance, the *ASV* (AMMI), *GGE*, and *WAASB* methods are assigned to group II, indicating simultaneous selection for agronomic performance and yield stability. However, for FRY, which shows higher variance of genotype-by-environment interaction (29.47%) compared to DMC (13.83%), *ASV*, *GGE*, and *WAASB* methods are categorized as static stability, while dynamic stability is associated with DMC. Therefore, the performance of FRY is penalized due to the high heterogeneity among the genotypes (resulting from a strong environmental effect, as indicated by the dispersion of methods in the biplot, and a.

${CV}_{g}$/${CV}_{r}$ ratio < 1, unfavorable for selection), which is not the case for DMC. In fact, dynamic stability in cassava has been reported by other researchers (Tumuhimbise et al. 2014; Chipeta et al. 2017).

The hypothesis raised by Flores et al. (1998) suggests that simultaneous selection methods tend to correlate in group 2, which serves as an intermediate group between group 1 (yield) and group 3 (stability). This hypothesis has been confirmed by other studies in wheat and rice, showing similar patterns of correlation between simultaneous selection methods and stability methods (Verma et al. 2020; Bornhofen et al. 2017; Sharifi et al. 2021). Genotypes allocated in groups 2 and 3 tend to exhibit a consistent response to environmental variation without being responsive to environmental improvements. This makes them suitable for cultivation in regions where growing conditions may be less favorable for cassava production. However, when the main target of selection is yield, the accuracy of selection based on these stability methods may be lower because they are more related to the static concept of stability (Becker and Leon 1988).

Based on the methods allocated in groups 2 and 3, genotypes BR11-24–156 and BR11-34–64 could be recommended for cultivation in farms with low use of agricultural inputs and technology. These genotypes exhibit competitive yields under such conditions (24.64 and 26.53 t ha^−1^ of FRY, respectively), but they do not respond significantly to environmental improvements. This recommendation takes into account parameters such as regression deviation (${S}^{2}d$), root mean square error ($RMSE$), and regression coefficient of determination (${R}^{2}$) (Eberhart and Russell 1966). However, it is important to note that all these genotypes were associated with the dynamic concept of stability for DMC, despite the formation of distinct groups.

It is important to note that both the concept of stability and the correlation between stability and adaptability methods may vary depending on the specific characteristic being evaluated. However, utilizing methods with diverse approaches and forming groupings provides greater confidence in selection, which should align with the objectives of the breeding program. It is worth mentioning that methods that quantify both stability and yield simultaneously tend to yield better results than those that solely provide information about stability.

Most parametric methods require the assumption of homogeneity of variance for analyzing genotype-by-environment interaction. In contrast, nonparametric methods are not bound by this assumption and therefore tend to reduce bias caused by outliers present in multi-environment trials (METs). Nonparametric methods also have little to no dependence on the distribution of the data, making them easily interpretable and complementary to use (Agustina et al. 2020). However, nonparametric models have limitations in multivariate analyses, and implementing biplot plots for better visualization of G × E interaction can be challenging (Agyeman et al. 2015).

The utilization of stability methods allocated in group I, such as $HMGV$ and ${Si}^{6}$, can assist cassava breeders in selecting the best genotypes in VCU trials for subsequent cultivation recommendations. On the other hand, methods more closely related to static stability can be employed in the selection of parents for breeding crosses (Kadhem et al. 2010). Considering the hypothesis that variance between genotypes can impact the discriminant power of the methods, both $Pi$ and ${Si}^{6}$ can be used when there is greater heterogeneity of variance among genotypes and environments. Conversely, parameters such as ${S}^{2}di, {R}^{2} and RMSE$, are more suitable when the variance is of low magnitude, as they provide a concise and reliable separation between biological and agronomic stability.

### Selection via multivariate index AMMI x GGE x WAASB x HMGV for performance, stability, and adaptability

An important strategy in breeding programs to address differences in genotype ratings is to explore stability and broad or specific adaptability, considering the significant nature of G × E interaction, through parametric (uni- and multivariate) and nonparametric approaches (Nassar and Huehn 1987). The combined use of these approaches has been widely explored in breeding programs due to their unique characteristics and benefits, as well as the graphical tools that facilitate efficient visualization of G × E.

Among the multivariate models, the AMMI is one of the main statistical tools for understanding G × E interaction. It separates and explores the main effects (additive variance) from the interaction (multiplicative) and identifies specific adaptations based on the magnitude and signs of the scores. Stable genotypes and environments are characterized by scores near zero on the IPCA1 and IPCA2 axes (Gauch and Zobel 1996, Gauch Jr 2013; Silva and Benin 2012). On the other hand, the GGE method removes the effect of the environment and expresses the response solely as a function of the genetic effect (G) and G × E interaction. This methodology allows the identification of broad and/or specific adaptations based on the distance of the genotype (or environment) from the origin of the biplot. It also considers the existence of mega-environments, which are sets of environments that share similar soil and climate characteristics. The discrimination and representativeness of the test environments are indicated by the size and angle of the vectors (Yan and Tinker 2006).

Both the AMMI and GGE methods can explore variance and genetic correlations between pairs of genotypes and environments. They aim to distinguish the pattern of G × E interaction from random error and have proven to be efficient in capturing a high proportion of the SQ_G×E_ (> 40%). They have also successfully identified genotypes that are stable and adaptable to different environments. However, in the present study, the GGE method explained a higher proportion of the total variance for FRY (75.27%) and DMC (90.66%) compared to the AMMI method, which captured 53.53% and 59.21% of the variance, respectively. Similar results were reported by Agyeman et al. (2015) when evaluating 10 cassava genotypes in six environments. These differences in explained variance may be attributed to: i) the lack of the inner product property of vectors in AMMI, ii) the fact that GGE does not directly explore the main effects, which may increase the influence of noise in the model due to G × E, resulting in a higher proportion of the SQ, or iii) the higher accuracy of the GGE model in detecting patterns of G × E (Agyeman et al. 2015; Yan 2016).

Previous multi-environment trials, both in the clonal selection phase of evaluation and VCU trials, have also reported higher sum-of-squares uptake in the GGE methodology (85%) compared to AMMI (72%) (Esuma et al. 2016; Rad et al. 2013). Considering simultaneous selection with G × E tending to zero, the AMMI and GGE methods identified the genotypes BR11-34–69 and BRS Novo Horizonte, respectively, as having the highest FRY and stability. Similarly, the BRS Novo Horizonte genotype showed the highest DMC and stability according to both the AMMI and GGE models, with Corrente also exhibiting similar characteristics according to the GGE method. However, it should be noted that a higher explanation of SQ_G×E_ does not always confer superiority to the GGE method, as a greater capture of total variance does not necessarily represent higher accuracy in the selection of superior genotypes due to the presence of noise in the model (Kvitschal et al. 2009; Yan 2016).

In this study, equal weights (50%) were assigned to average performance and stability for both FRY and DMC by using the *WAASB* index. The *WAASB* index offers several advantages: it is based on mixed models, allowing flexibility in choosing economic weights based on breeders' interests; it enables the exploration of G × E interaction patterns using more PC; and it exhibits lower sensitivity to outliers inherent in METs trials (Olivoto et al. 2019). In the biplot, genotypes and environments are grouped into four categories: unproductive and unstable genotypes (Group 1), productive and unstable genotypes (Group 2, characterized by high G × E and discriminative environments), low yield (Group 3), and high yield (Group 4, with lower *WAASB* value, broad adaptation, and low discriminative ability) (Olivoto et al. 2019). In this scenario, BRS Novo Horizonte stood out in Group 4, as did the PP-2018 environment for both traits.

Lastly, the *HMGV* based on BLUPs offers several advantages, including greater flexibility in dealing with heterogeneity of variances and covariances between environments, elimination of G × E noise, results generated on the scale of the characteristic itself, and taking into account heritability in the estimation of the model with simultaneous selection for performance and genotypic stability. Therefore, when a genotype exhibits small deviations within and between sites, it is considered stable. Thus, *HMGV* highlighted the genotype BR11-34–41 and BRS Novo Horizonte as the most important genotypes for increasing FRY and DMC, respectively.

### Characterization of contrasting environments and mega-environments via GGE

Environments vary in terms of the quality and quantity of resources available to plants, as well as climatic conditions, leading to different phenotypic expressions of genotypes due to unpredictable environmental factors (environmental covariates). Quantifying and exploring environmental variations are crucial for determining the performance profile of a genotype, as the realization of its productive potential depends on meeting ideal soil and climatic conditions (Malosetti et al. 2013). Identifying and classifying genotypes and environments suitable for cultivation is of fundamental importance in any breeding program, as it guides the regionalized recommendation of the best genotypes for target environments where inferences and predictions are valid and accurate (Van Eeuwijk et al. 2016).

In general, optimal environments should effectively discriminate genotypic effects while also being representative of mega-environments. Mega-environments are defined as growing regions with reasonably homogeneous characteristics, but they don't necessarily have to be contiguous areas that produce similar performance in genotypes (Yan and Rajcan 2002; Yan 2016). Within each mega-environment, the effects of G × E interaction are limited or not significant, indicating similar soil and climate conditions that lead to comparable phenotypic responses in groups of genotypes with angles smaller than 90° (within the mega-environment). Angles greater than 90° indicate uncorrelated environments, and angles greater than 180° indicate negative correlations associated with distinct edaphoclimatic conditions. For example, a difference in performance was observed between UFRB-2019 and NR-2019 for FRY, although more than 70% of the environments showed a high correlation with each other (Yan 2016).

Climatic conditions tend to have a greater influence on the phenotypic expression of genotypes compared to soil conditions. This can be observed in the environments UFRB-2019 and NR-2019, which had distinct responses between traits, with long vectors in the AMMI biplot for FRY (indicating high instability) and short vectors in the GGE biplot for DMC (indicating high stability). Despite this, there was high homogeneity among environmental covariates such as temperature and precipitation, resulting in a high correlation among environments with weak or no G × E patterns (Fig. 1). In sorghum, Oliveira et al. (2020b) reported that historical climate data can guide the recommendation and classification of target genotypes and environments, as agronomic performance is affected by precipitation and temperature, leading to changes in classification.

The perpendicular lines drawn from the origin of the biplot in the mega-environments divide the polygon into sectors. Genotypes located at the vertices of the polygon can be the best performers if they are associated with one or more environments in that sector, or the worst performers if they have no environments in that sector (Yan 2016; Van Eeuwijk et al. 2016). When all evaluated environments fall within the same sector, it indicates that a single genotype performs well in all environments. For example, BRS Novo Horizonte was allocated at the vertex with the highest DMC values in all environments and can be considered a reference genotype for evaluation. Similar findings were reported by Adetoro et al. (2021), where DMC showed five sectors in all environments, and only one mega-environment was identified, demonstrating the stability and high performance of this genotype and indicating a high correlation between environments.

Indeed, if the environments are located in distinct sectors in the biplot, it suggests the presence of more than one genotype with high yield in different environments. This is exemplified by the FRY trait, where two mega-environments were formed, and the genotype BR11-34–41 exhibited the highest root productivities in a total of nine environments. For instance, NR-2019 and UFRB-2018 showed long vectors (indicating high G × E interaction) and short vectors (indicating low G × E), respectively, demonstrating their discriminative and representative capacities (Yan 2016; Van Eeuwijk et al. 2016).

Recommendation of a genotype that combines high agronomic performance across multiple variables with stability and adaptability is a complex task. However, the present study indicates that the new cassava genotypes outperformed the control genotypes in the face of environmental changes. It is important to further explore the repeatability, reliability, and suitability of different methods to establish alternative approaches that ensure the broad applicability of these methods in multi-environment trials.

### Future perspectives

The primary objective of the breeding program is to identify genotypes with superior performance and increased phenotypic stability. However, the METs trials exhibit an inherent characteristic of having an imbalanced number of genotypes evaluated across different years. This imbalance occurs due to the exclusion of genotypes with inferior performance and the inclusion of new clones in subsequent crop years. Consequently, traditional fixed effect methods demonstrate limited efficacy in such situations. To address this, the exploration of G × E interaction becomes more accurate by employing mixed models. Modern approaches, like the *MPS* and *MTMPS* indices (Olivoto et al. 2019), offer improved precision by considering both performance and stability across multiple traits. These indices are particularly valuable for identifying superior genotypes that exhibit desirable agronomic characteristics such as root nutritional quality and disease resistance. By incorporating diverse stability indices, these models contribute to the identification of genotypes that excel in various traits of agronomic interest. Such advancements aim to enhance the efficiency of recommending new genotypes while better understanding the influence of environmental factors, especially for complex traits with substantial environmental impact, such as FRY.

## Conclusion

The recommendation of genotypes that exhibit high yield, stability, and adaptability across multiple traits is indeed a complex task due to the nature and significance of the G × E interaction. However, even in the final validation phase of genotypes, this study was successful in selecting genotypes with high agronomic performance and potential for improvement through selection.

The genetic parameters indicated that the best genotypes (BR11-34–41 and BR11-34–69) had the potential to achieve yields greater than 32 t ha^−1^and genotypes BR12-107–002 and BR11-24–156 had high dry matter content in the roots (> 37%). These genotypes, along with BRS Novo Horizonte, outperformed the reference varieties. Different methods used in the study showed that 72% identified BRS Novo Horizonte as the genotype with the highest stability and performance for dry matter content, while 47% identified BR11-34–41 and BR11-34–69 as potential genotypes for commercial release with high root yield and intermediate dry matter content.

The positive and highly significant correlations between parametric and non-parametric methods provided a better understanding of their ability to exploit the G × E. Four groups were formed based on the stability concept, with the first group highly correlated with the traits under analysis (agronomic stability). The genotype BR11-24–156 exhibited high static stability according to 50% of the methods (groups II, III, and IV), although it may not be recommended for favorable environments due to its low yield in those conditions. However, it can still be used as a parent in the breeding program. The multivariate approaches (AMMI, *GGE*, and *WAASB*) were efficient and complementary in studying the G × E and simultaneous selection. The genotype BR11-24–156 exhibited static stability and BRS Novo Horizonte showed dynamic stability for root yield. For dry matter content, the genotypes BR11-34–69 and BR12-107–002 exhibited static stability, while BRS Novo Horizonte showed dynamic stability. Therefore, these methods can be used in a complementary and simultaneous manner to select genotypes, thereby increasing the reliability of the breeding program in recommending superior genotypes for cassava genetic improvement.

### Supplementary Information

Below is the link to the electronic supplementary material.

Summary of phenotypic means for (A) fresh root yield (t ha-1) and (B) dry matter content (%) of 12 cassava genotypes in 12 different environments. (TIF 168 KB)

Biplots for: A) fresh root yield (FRY, t ha-1) and B) dry matter content (DMC, %), versus weighted mean of the absolute scores for the best unbiased linear predictions of the genotype versus environment interaction (WAASB), obtained from the evaluation of 12 cassava genotypes in 12 environments (TIF 1214 KB)

## References

[CR1] Adetoro NA, Oworu OO, Nassir AL, Belo A, Parkes E, Ogunbayo SA, Akinwale MG, Aina OO, Afolabi A, Iluebbey P, Sanni LO, Maziya-Dixo B, Dixon A, Kulakow P (2021) Evaluation of improved cassava genotypes for yield and related traits for a better breeding strategy under different agroecologies in Nigeria. Euphytica 217:73. 10.1007/s10681-021-02798-9 10.1007/s10681-021-02798-9

[CR2] Adjebeng-Danquah J, Manu-Aduening J, Gracen VE, Asante IK, Offei SK (2017) AMMI stability analysis and estimation of genetic parameters for growth and yield components in cassava in the forest and Guinea Savannah ecologies of Ghana. Int J Agron 2017:1–10. 10.1155/2017/8075846 10.1155/2017/8075846

[CR3] Agustina AF, Khumaida N, Sintho Wahyuning Ardie WS, Muhamad S (2020) Nonparametric stability analysis of starch content of gamma irradiated cassava at three locations in West Java Indonesia. J Trop Crop Sci 7(2):66–74. 10.29244/jtcs.7.02.66-74 10.29244/jtcs.7.02.66-74

[CR4] Agyeman A, Parkes E, Peprah B (2015) AMMI and GGE biplot analyses of root yield performance of cassava genotypes in forest and coastal ecologies. Int J Agric Pol Res 3:122–132. 10.15739/IJAPR.034 10.15739/IJAPR.034

[CR5] Akinwale MG, Akinyele BO, Dixon AGO, Odiyi AC (2010) Genetic variability among forty-three cassava genotypes in three agro-ecological zones of Nigeria. J Plant Breed Crop Sci 2(5):104–109. 10.5897/JPBCS.9000065 10.5897/JPBCS.9000065

[CR6] Akinwale MG, Akinyele BO, Odiyi AC, Dixon AGO (2011) Genotype x environment interaction and yield performance of 43 improved cassava (*Manihot esculenta* Crantz) genotypes at three agro-climatic Zones in Nigeria. Br Biotechnol J 1(3):68–84 10.9734/BBJ/2011/475

[CR7] Alwala S, Kwolek T, Mcpherson M, Pellow J, Meyer DA (2010) Comprehensive comparison between Eberhart and Russell joint regression and GGE biplot analyses to identify stable and high yielding maize hybrids. Field Crops Res 119:225–230. 10.1016/j.fcr.2010.07.010 10.1016/j.fcr.2010.07.010

[CR8] Andrade LRB, Sousa MB, Oliveira EJ, Resende MDV, Azevedo CF (2019) Cassava yield traits predicted by genomic selection methods. PLoS ONE 14:11. 10.1371/journal.pone.0224920 10.1371/journal.pone.0224920 PMC6855463 31725759

[CR9] Annicchiarico P (1992) Cultivar adaptation and recommendation from alfalfa trials in Northern Italy. J Genet Plant Breed 46(3):269–278

[CR10] Arnhold E (2013) Package in the R environment for analysis of variance and complementary analyses. Braz J Vet Res Anim Sci 50(6):488–492. 10.11606/issn.1678-4456.v50i6p488-492 10.11606/issn.1678-4456.v50i6p488-492

[CR11] Becker HC, Leon J (1988) Stability analysis in plant breeding. Plant Breed 101(1):1–23. 10.1111/j.1439-0523.1988.tb00261.x 10.1111/j.1439-0523.1988.tb00261.x

[CR12] Bornhofen E, Benin G, Storck L, Woyann LG, Duarte T, Stoco MG, Marchioro SV (2017) Statistical methods to study adaptability and stability of wheat genotypes. Bragantia 76:1–10. 10.1590/1678-4499.557 10.1590/1678-4499.557

[CR13] Ceballos H, Kulakow P, Hershey C (2012) Cassava breeding: current status, bottlenecks and the potential of biotechnology tools. Trop Plant Biol 5:73–87. 10.1007/s12042-012-9094-9 10.1007/s12042-012-9094-9

[CR14] Ceballos H, Kawuki RS, Gracen VE, Yencho GC, Hershey CH (2015) Conventional breeding, marker assisted selection, genomic selection and inbreeding in clonally propagated crops: a case study for cassava. Theor Appl Genet 128:1647–1667. 10.1007/s00122-015-2555-4 26093610 10.1007/s00122-015-2555-4 PMC4540783

[CR15] Ceballos H, Rojanaridpiched C, Phumichai C, Becerra LA, Kittipadakul P, Iglesias C, Gracen VE (2020) Excellence in cassava breeding: perspectives for the future. Crop Breed Genet Genom 2(2):e200008

[CR16] Cepea - Center for Advanced Studies in Applied Economics - CEPEA-Esalq/USP (2022). Crop forecast data. https://www.cepea.esalq.usp.br/br/consultas-ao-banco-de-dados-do-site.aspx. Accessed 21 February 2022

[CR17] Chipeta MM, Melis RA, Shanahan PE, Sibiya J, Benesi IR (2017) Genotype x environment interaction and stability analysis of cassava genotypes at different harvest times. J Anim Plant Sci 27(3):901–919

[CR18] Dwamena HA, Tawiah K (2022) Kodua ASA (2022) The effect of rainfall, temperature, and relative humidity on the yield of cassava, yam, and maize in the ashanti region of Ghana. Int J Agron 1:1–12. 10.1155/2022/9077383 10.1155/2022/9077383

[CR19] Eberhart SA, Russell WA (1966) Stability parameters for comparing varieties. Crop Sci 6(1):36–40. 10.2135/cropsci1966.0011183X000600010011x 10.2135/cropsci1966.0011183X000600010011x

[CR20] El-Sharkawy MA (2007) Physiological characteristics of cassava tolerance to prolonged drought in the tropics: Implications for breeding cultivars adapted to seasonally dry and semiarid environments. Braz J Plant Physiol 19(4):257–286. 10.1590/S1677-04202007000400003 10.1590/S1677-04202007000400003

[CR21] El-Sharkawy MA (2012) Stress-tolerant cassava: the role of integrative ecophysiology-breeding research in crop improvement. Open J Soil Sci 2(2):162–186. 10.4236/ojss.2012.22022 10.4236/ojss.2012.22022

[CR22] Esuma W, Kawuki RS, Herselman L, Labuschagne MT (2016) Stability and genotype by environment interaction of provitamin A carotenoid and dry matter content in cassava in Uganda. Breed Sci 66(3):434–443 27436954 10.1270/jsbbs.16004 PMC4902464

[CR23] Ewa F, Nwofia E, Egesi C, Olasanmi B, Okogbenin E (2017) Genetic variability, heritability and variance components of some yield and yield related traits in second backcross population (BC2) of cassava. Afr J Plant Sci 11(6):185–189. 10.5897/AJPS2015.1324 10.5897/AJPS2015.1324

[CR24] Faostat. Food and Agricultural Organization of the United Nations Online Statistics Database. 2020. Available in: http://www.fao.org/faostat/en/#data/FBS, Accessed 21 February 2022.

[CR25] Finlay KW, Wilkinson GN (1963) The analysis of adaptation in a plant-breeding programmer. Aust J Agric Res 14(6):742–754. 10.1071/AR9630742 10.1071/AR9630742

[CR26] Flores F, Moreno MT, Cubero JI (1998) A comparison of univariate and multivariate methods to analyze G×E interaction. Field Crops Res 56(3):271–286. 10.1016/S0378-4290(97)00095-6 10.1016/S0378-4290(97)00095-6

[CR27] Fotso AP, Hanna R, Kulakow P, Parkes E, Iluebbey PO, Ngome FA, Suh C, Massussi J, Choutnji I, Wirnkar VL (2018) AMMI analysis of cassava response to contrasting environments: case study of genotype by environment effect on pests and diseases, root yield, and carotenoids content in Cameroon. Euphytica 214(9):155–167. 10.1007/s10681-018-2234-z 10.1007/s10681-018-2234-z

[CR28] Frutos E, Galindo MP, Leiva V (2013) An interactive biplot implementation in R for modeling genotype-by-environment interaction. Stochastic Environ Res Risk Assess 28(7):1629–1641. 10.1007/s00477-013-0821-z 10.1007/s00477-013-0821-z

[CR29] Gauch HG Jr (2013) A simple protocol for AMMI analysis of yield trials. Crop Sci 53(5):1860–1869. 10.2135/cropsci2013.04.0241 10.2135/cropsci2013.04.0241

[CR30] Gauch HG, Zobel RW (1996) AMMI analysis of yield trials. In: Kang MS, Gauch HG (eds) Genotype by environment interaction. CRC Press, Boca Raton, pp 85–122

[CR31] Halsey ME, Olsen KM, Taylor NJ, Chavarriaga-Aguirre P (2008) Reproductive biology of cassava and Isolation of experimental field trials. Crop Sci 48(1):49–59. 10.2135/cropsci2007.05.0279 10.2135/cropsci2007.05.0279

[CR32] IBGE - Brazilian Institute of Geography and Statistics (2021). Crop forecast data, Systematic Survey of Agricultural Production: cassava. https://sidra.ibge.gov.br/home/pnadcm. Accessed 26 February 2022.

[CR33] Inmet - National Institute of Meteorology (2018). BDMEP - Meteorological Database for Teaching and Research - Historical Series - Monthly Data – Average Wind Speed (mps). Brasilia. http://www.inmet.gov.br/portal/index.php?r=bdmep/bdmep. Accessed 15 May 2022

[CR34] Jiwuba L, Danquah A, Asante I, Blay E, Onyeka J, Danquah E, Egesi C (2020) Genotype by environment interaction on resistance to cassava green mite associated traits and effects on yield performance of cassava genotypes in Nigeria. Front Plant Sci 11:572200. 10.3389/fpls.2020.572200 33013995 10.3389/fpls.2020.572200 PMC7498573

[CR35] Kadhem FA, Al-Nedawi IS, Al-Atabe SD, Baktash FY, Jour DAS (2010) Association between parametric and nonparametric measures of phenotypic stability in Rice genotypes (*Oryza**sativa* L.). Diyala Agric Sci J 2(2):20–33

[CR36] Kawano K, Fukuda WMG, Cenpukdee U (1987) Genetic and environmental effects on dry matter content of cassava root. Crop Sci 27(1):69–74. 10.2135/cropsci1987.0011183X002700010018x 10.2135/cropsci1987.0011183X002700010018x

[CR37] Kundy A, Mkamilo GS, Misangu RN (2015) Genetic variability among six traits in twelve cassava (*Manihot esculenta* Crantz) genotypes in Southern Tanzania. J Nat Sci Res 5(12):33–38

[CR38] Kvitschal MV, Vidigal Filho PS, Scapim CA, Gonçalves-Vidigal MC, Sagrilo E, Pequeno MG, Rimoldi F (2009) Comparison of methods for phenotypic stability analysis of cassava (*Manihot esculenta* Crantz) genotypes for yield and storage root dry matter content. Braz Arch Biol Technol 52(1):163–175. 10.1590/S1516-89132009000100022 10.1590/S1516-89132009000100022

[CR39] Lin CS, Binns MR (1988) A superiority measure of cultivar performance for cultivar x location data. Can J Plant Sci 68(1):193–198. 10.4141/cjps88-018 10.4141/cjps88-018

[CR40] Malosetti M, Ribaut JM, Van Eeuwijk FA (2013) The statistical analysis of multi-environment data: modeling genotype-by-environment interaction and its genetic basis. Front Physiol 4:44. 10.3389/fphys.2013.00044 23487515 10.3389/fphys.2013.00044 PMC3594989

[CR41] Maroya NG, Kulakow P, Dixon AGO, Maziya-Dixon BB (2012) Genotype × environment interaction of mosaic disease, root yields and total carotene concentration of yellow-fleshed cassava in Nigeria. Int J Agron 2012:1–8. 10.1155/2012/434675 10.1155/2012/434675

[CR42] Masinde EA, Mkamillo G, Ogendo JO, Hillocks R, Mulwa RMS, Kimata B, Maruthi MN (2018) Genotype by environment interactions in identifying cassava (*Manihot esculenta* Crantz) resistant to cassava brown streak disease. Field Crops Res 215:39–48 10.1016/j.fcr.2017.10.001

[CR43] Mendiburu F (2021) Statistical Procedures for Agricultural Research. Package ‘*Agricolae*,’ version 1.3.3 Comprehensive R Archive Network, Institute for Statistics and Mathematics. http://cran.r-project.org/web/packages/agricolae/agricolae.pdf.

[CR44] Mtunguja M, Laswai H, Kanju EE, Ndunguru J (2016) Effect of genotype and genotype by environment interaction on total cyanide content, fresh root, and starch yield in farmer-preferred cassava landraces in Tanzania. Food Sci Nutr 4(6):791–801. 10.1002/fsn3.345 27826428 10.1002/fsn3.345 PMC5090642

[CR45] Nassar R, Huehn M (1987) Studies on estimation of phenotypic stability: tests of significance for non- parametric measures of phenotypic stability. Biometrics 43(1):45–53. 10.2307/2531947 10.2307/2531947

[CR46] Nduwumuremyi A, Melis R, Shanahan P, Theodore A (2017) Interaction of genotype and environment effects on important traits of cassava (*Manihot esculenta* Crantz). Crop J 5(5):373–386 10.1016/j.cj.2017.02.004

[CR47] Noerwijati K, Taryono N, Prajitno D (2014) Fresh tuber yield stability analysis of fifteen cassava genotypes across five environments in East Java (Indonesia) using GGE biplot. Energy Procedia 47:156–165. 10.1016/j.egypro.2014.01.209 10.1016/j.egypro.2014.01.209

[CR48] Oliveira EJ, Santana FA, Oliveira LA, Santos VS (2014) Genetic parameters and prediction of genotypic values for root quality traits in cassava using REML/BLUP. Genet Mol Res 13(3):6683–6700. 10.4238/2014 25177949 10.4238/2014

[CR49] Oliveira EJ, Aidar ST, Morgante CV, Chaves ARM, Cruz JL, Coelho Filho MA (2015) Genetic parameters for drought tolerance in cassava. Pesqui Agropecu Bras 50(3):233–241. 10.1590/S0100-204X2015000300007 10.1590/S0100-204X2015000300007

[CR50] Oliveira EJ, Fukuda WMG, Oliveira SAS, Ringenberg R, Silva MR, Souza AS, Silva LLA, Oliveira MLF, Silva SRJ (2020a) BRS Novo Horizonte - a new cassava variety for industrial use. Crop Breed Appl Technol 20:2. 10.1590/1984-70332020v20n2c24 10.1590/1984-70332020v20n2c24

[CR51] Oliveira ICM, Guilhen JHS, Ribeiro PCO, Gezan SA, Schaffert RE, Simeone MLF, Damasceno CMB, Carneiro JES, Carneiro PCS, Parrella RAC, Pastina MM (2020b) Genotype-by-environment interaction and yield stability analysis of biomass sorghum hybrids using factor analytic models and environmental covariates. Field Crops Res 257:107929. 10.1016/j.fcr.2020.107929 10.1016/j.fcr.2020.107929

[CR52] Olivoto T (2020) Lúcio AD (2020) Metan: R package for multi-environment trial analysis. Met Eco Evo 11(6):783–789. 10.1111/2041-210X.13384 10.1111/2041-210X.13384

[CR53] Olivoto T, Lúcio ADC, Silva JAG, Marchioro VS, Souza VQ, Jost E (2019) Mean performance and stability in multi-environment trials i: combining features of AMMI and BLUP techniques. Agron J 111(6):2949–2960. 10.2134/agronj2019.03.0220 10.2134/agronj2019.03.0220

[CR54] Peprah BB, Parkes E, Manu-Aduening J, Kulakow P, Van Biljon A, Labuschagne M (2020) Genetic variability, stability and heritability for quality and yield characteristics in provitamin A cassava variety. Euphytica 216(31):1–13. 10.1007/s10681-020-2562-7 10.1007/s10681-020-2562-7 PMC6988135 32055054

[CR55] Perkins JM, Jinks J (1968) Environmental and genotype-environmental components of variability III. Multiple Lines and Crosses Heredity 23(3):339–356. 10.1038/hdy.1968.48 5250121 10.1038/hdy.1968.48

[CR56] Pimentel-Gomes F (2009) Experimental statistics course, 15th edn. FEALQ, Piracicaba, SP

[CR57] Purchase J, Hatting H, Van Deventer C (2000) Genotype × environment interaction of winter wheat (*Triticum**aestivum* L.) in South Africa: II. Stability analysis of yield performance. S Afr J Plant Soil 17(3):101–107 10.1080/02571862.2000.10634878

[CR58] R Core Team. R: A language and environment for statistical computing. R Foundation for Statistical Computing, Vienna, 2022. Disponível em: https://cran.r-project.org/bin/windows/base/

[CR59] Rad MRN, Kadir MA, Rafii MY, Jaafar HZE, Naghavi MR, Ahmadi F (2013) Genotype x environment interaction by AMMI and GGE biplot analysis in three consecutive generations of wheat (*Triticum aestivum*) under normal and drought stress conditions. Aust J Crop Sci 7(7):956–961

[CR60] Sharifi P, Ebadi AA, Hallajian MT (2021) Stability of rice mutant lines by linear mixed model and suggestion a new index based for yield performance and stability, Res Sq, 10.21203/rs.3.rs-895572/v1

[CR61] Silva RR, Benin G (2012) Biplot analysis: concepts, interpretations and uses. Cienc Rural 42(8):1404–1412. 10.1590/S0103-84782012000800012 10.1590/S0103-84782012000800012

[CR62] Singh RK, Chaudhary BD (1979) Biometrical Methods in Quantitative Genetic Analysis, Kalyani publishers/Lyall Bk Depot, 304.

[CR63] Souza LD, Souza LS, Gomes JC (2006) Edaphic Requirements of Cassava Culture In: Souza LS, Farias ARN, Mattos PLP, Fukuda WMG (Eds). Socioeconomic and agronomic aspects of manioc. Cruz das Almas: Embrapa Cassava and Tropical Fruits, pp 170–214.

[CR64] Ssemakula GN, Dixon A (2007) Genotype x environment interaction, stability and agronomic performance of carotenoid-rich cassava clones. Sci Res Essays 2(9):390–399

[CR65] Stapleton G (2012) Global starch market outlook and competing starch raw materials for by product segment and region. Pricing Outlook and Cassava Growth Potential. Cassava Starch World 2010. Centre for Management Technology (CMT), Phnom Penh.

[CR66] Tironi LF, Alves AF, Zanon AJ, Freitas CPO, Santos ATL, Cardoso P, Tonel,GP, Rodrigues LB, Tagliapietra BL, Silva MN, Streck NA (2019) Ecofisiologia da mandioca visando altas produtividades, Editora GR, Santa Maria.

[CR67] Tumuhimbise R, Melis R, Shanahan P, Kawuki R (2014) Genotype × environment interaction effects on early fresh storage root yield and related traits in cassava. Crop J 2(5):329–337. 10.1016/j.cj.2014.04.008 10.1016/j.cj.2014.04.008

[CR68] Van Eeuwijk FA, Bustos-Korts DV, Malosetti M (2016) What should students in plant breeding know about the statistical aspects of genotype × environment interactions? Crop Sci 56(5):2119–2140 10.2135/cropsci2015.06.0375

[CR69] Verma A, Singh GP (2020) Stability index based on weighted average of absolute scores of AMMI and yield of wheat genotypes evaluated under restricted irrigated conditions for Peninsular Zone. Int J Environ Agric Biotechnol 13(4):371–381

[CR70] Wolfe MD, Kulakow P, Rabbi IY, Jannink JL (2016) Marker-based estimates reveal significant nonadditive effects in clonally propagated cassava (*Manihot**esculenta*): implications for the prediction of total genetic value and the selection of varieties. G3(bethesda) 6(11):3497–3506 27587297 10.1534/g3.116.033332 PMC5100848

[CR71] Wolfe MD, Del Carpio DP, Alabi O, Ezenwaka LC, Ikeogu UN, Kayondo IS, Jannink JL (2017) Prospects for genomic selection in cassava breeding. Plant Genome 10:3 10.3835/plantgenome2017.03.0015 PMC7822052 29293806

[CR72] Wricke G (1962) Übereine Methode zür Erfassung der Okologischen Streubreite em Feldresuchen. Z Pflanzenzuchtg 47:92–96

[CR73] Yan W (2016) Analysis and handling of G×E in a practical breeding program. Crop Sci 56(5):2106–2118. 10.2135/cropsci2015.06.0336 10.2135/cropsci2015.06.0336

[CR74] Yan W, Holland JB (2010) A heritability-adjusted GGE biplot for test environment evaluation. Euphytica 171(3):355–369. 10.1007/s10681-009-0030-5 10.1007/s10681-009-0030-5

[CR75] Yan WK, Rajcan I (2002) Biplot analysis of test sites and trait relations of soybean in Ontario. Crop Sci 42(1):11–20. 10.2135/CROPSCI2002.1100 11756248 10.2135/CROPSCI2002.1100

[CR76] Yan W, Tinker A (2006) Biplot analysis of multi environment trial data: principles and applications. Can J Plant Sci 86(3):623–645. 10.4141/P05-169 10.4141/P05-169

