# Supplementary figures and images for: Stability and genetic parameters for cassava yield attributes in the tropical humid region of Brazil
Source: Euphytica. 2024 Jul 19;220(8):127. doi: 10.1007/s10681-024-03384-5 (PMC11271428; doi:10.1007/s10681-024-03384-5)

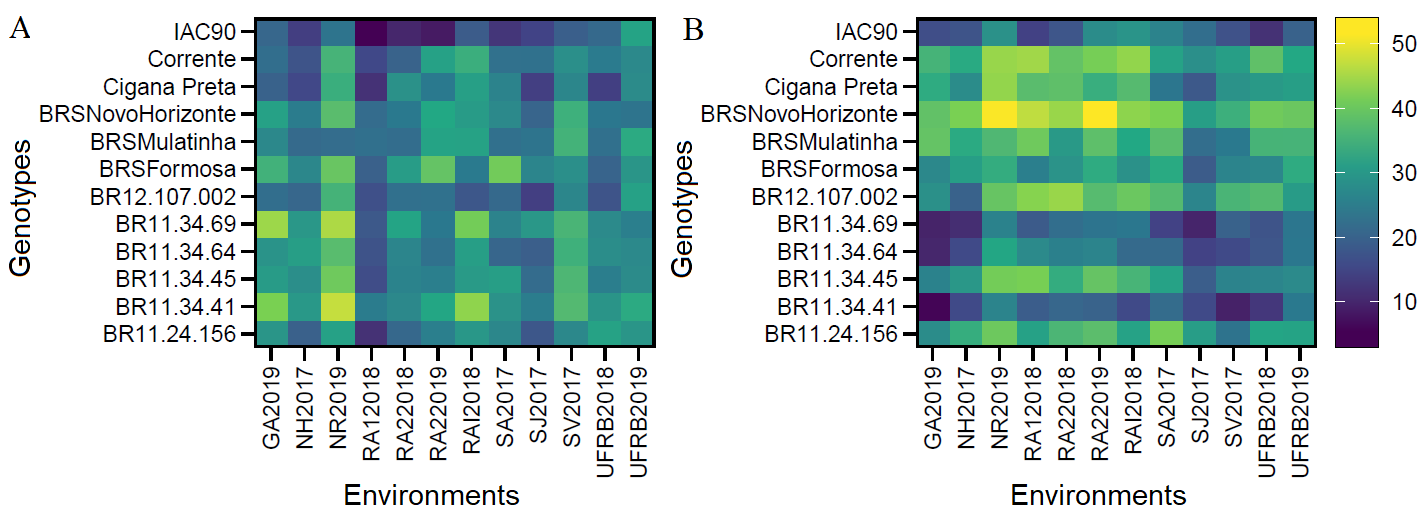

Supplement: Supplementary file 1 — Summary of phenotypic means for (A) fresh root yield (t ha-1) and (B) dry matter content (%) of 12 cassava genotypes in 12 different environments. (TIF 168 KB) [file 10681_2024_3384_MOESM1_ESM.tif]

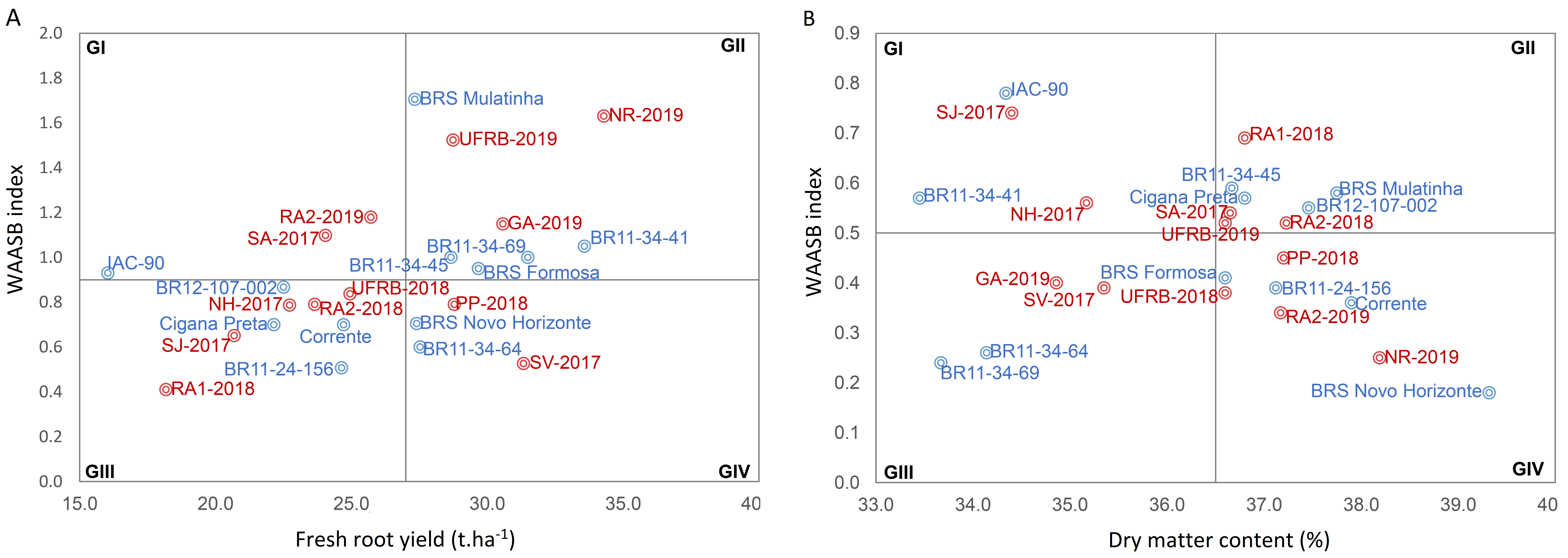

Supplement: Supplementary file 2 — Biplots for: A) fresh root yield (FRY, t ha-1) and B) dry matter content (DMC, %), versus weighted mean of the absolute scores for the best unbiased linear predictions of the genotype versus environment interaction (WAASB), obtained from the evaluation of 12 cassava genotypes in 12 environments (TIF 1214 KB) [file 10681_2024_3384_MOESM2_ESM.tif]
